# Swirls and scoops: Ice base melt revealed by multibeam imagery of an Antarctic ice shelf

**DOI:** 10.1126/sciadv.adn9188

**Published:** 2024-07-31

**Authors:** Anna Wåhlin, Karen E. Alley, Carolyn Begeman, Øyvind Hegrenæs, Xiaohan Yuan, Alastair G. C. Graham, Kelly Hogan, Peter E. D. Davis, Tiago S. Dotto, Clare Eayrs, Robert A. Hall, David M. Holland, Tae Wan Kim, Robert D. Larter, Li Ling, Atsuhiro Muto, Erin C. Pettit, Britney E. Schmidt, Tasha Snow, Filip Stedt, Peter M. Washam, Stina Wahlgren, Christian Wild, Julia Wellner, Yixi Zheng, Karen J. Heywood

**Affiliations:** ^1^ Department of Marine Sciences, University of Gothenburg, Gothenburg, Sweden.; ^2^ Centre for Earth Observation Science, University of Manitoba, Winnipeg, Canada.; ^3^ Fluid Dynamics and Solid Mechanics, Los Alamos National Laboratory, Los Alamos, NM, USA.; ^4^ Department of Uncrewed Platforms, Kongsberg Discovery, Horten, Norway.; ^5^ College of Surveying and Geo-Informatics, Tongji University, Shanghai, China.; ^6^ School of Earth and Environmental Sciences, Cardiff University, Main Building, Park Place, Cardiff CF10 3AT, Wales, UK.; ^7^ British Antarctic Survey, Cambridge, UK.; ^8^ National Oceanography Centre, Southampton, UK.; ^9^ Korea Polar Research Institute, Incheon, Republic of Korea.; ^10^ Centre for Ocean and Atmospheric Sciences, School of Environmental Sciences, University of East Anglia, Norwich, UK.; ^11^ Environmental Fluid Dynamics Laboratory, New York University, New York, NY, USA.; ^12^ Division of Robotics, Perception and Learning, KTH Royal Institute of Technology, Stockholm, Sweden.; ^13^ Department of Earth and Environmental Science, Temple University, Philadelphia, PA, USA.; ^14^ College of Earth, Ocean, and Atmospheric Sciences, Oregon State University, Corvallis, OR, USA.; ^15^ Departments of Astronomy and of Earth and Atmospheric Sciences, Cornell University, Ithaca, NY, USA.; ^16^ Department of Geophysics, Colorado School of Mines, 1500 Illinois St, Golden, CO, USA.; ^17^ Department for Geoscience, University of Tübingen, Tübingen, Germany.; ^18^ Department of Earth and Atmospheric Sciences, University of Houston, Houston, TX, USA.

## Abstract

Knowledge gaps about how the ocean melts Antarctica’s ice shelves, borne from a lack of observations, lead to large uncertainties in sea level predictions. Using high-resolution maps of the underside of Dotson Ice Shelf, West Antarctica, we reveal the imprint that ice shelf basal melting leaves on the ice. Convection and intermittent warm water intrusions form widespread terraced features through slow melting in quiescent areas, while shear-driven turbulence rapidly melts smooth, eroded topographies in outflow areas, as well as enigmatic teardrop-shaped indentations that result from boundary-layer flow rotation. Full-thickness ice fractures, with bases modified by basal melting and convective processes, are observed throughout the area. This new wealth of processes, all active under a single ice shelf, must be considered to accurately predict future Antarctic ice shelf melt.

## Teaser

A unique dataset from beneath an Antarctic ice shelf shows a varied icescape created by differential melt mechanisms.

## INTRODUCTION

Changes in ocean temperature and circulation are driving mass loss from Antarctica through basal melting of floating ice shelves (*1*, *2*). Ice shelf thinning and break-up reduce buttressing forces that hold back grounded ice (*3*), which may initiate feedbacks associated with grounding-line retreat (*4*), accelerating global sea level rise (*5*).

Basal melt rates can vary by orders of magnitude beneath the same ice shelf (*6*–*10*) and are controlled by the rate at which warm, saline water is brought into contact with the ice base (*11*–*13*). Shear-driven turbulence is an efficient way to mix warm water into the ice-ocean boundary layer and forms the basis for subgridscale parameterizations of basal melt in many ocean models (*14*, *15*). Furthermore, vertical convection, including double-diffusive convection, can move heat to the ice base. This happens when warmer, saltier water underlies colder, fresher water and generally results in an order of magnitude lower melt rates than with shear turbulence (*16*–*21*).

Different basal melt processes are expected to form distinct ice base features. Ice shelf basal roughness correlates generally with melt rates across many ice shelves (*1*, *22*). Observations (*23*–*25*) suggest that steps or terraces are common on ice shelf bases and may form due to differences in basal melt rates tied to basal slope. Basal channels, stretching many kilometers (*10*, *26*, *27*), have been linked to enhanced basal melt (*28*). Elevated melt rates and decimeter-scale scallops have been observed on the steep sides of basal fractures (*25*, *29*–*31*). Analysis of these processes has been limited by a lack of data from the ice base, particularly high-resolution surveys and complementary datasets covering sufficiently large areas to understand the extent and representativeness of the features.

**Fig. 1.**
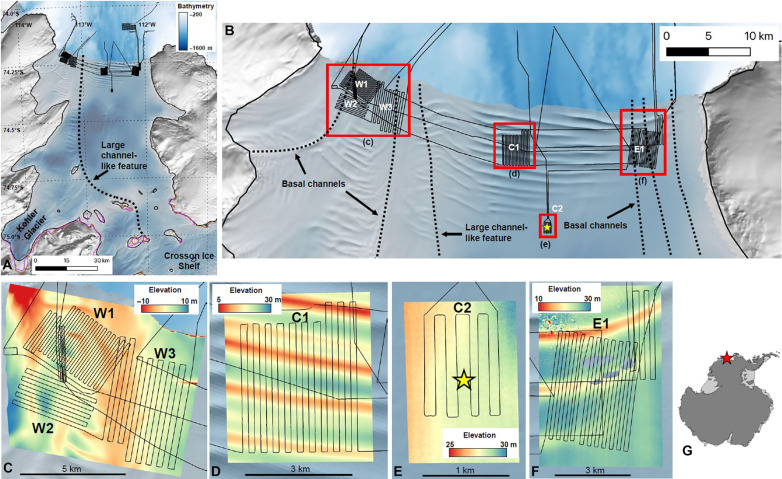
Dotson Ice Shelf. (**A** and **B**) Reference Elevation Model of Antarctica mosaic (*66*) of DIS with the regions and features discussed in the text labeled: basal channel features (dashed lines), ~2000 Landsat-derived grounding line [thick black lines; (*67*)], and 1992–2020 Interferometric Synthetic Aperture Radar–derived grounding lines [pink lines; (*68*, *69*)]. Thin black lines show the AUV mission paths, and red squares in (B) show the location of the zoomed-in areas in (**C**) to (**F**). Blue shades show bathymetry (*70*) [color bar in (A)]. [(C) to (F)] Zoomed-in areas showing the ice surface elevation at the survey areas, referenced to the WGS84 ellipsoid. The yellow star in (E) shows the location of the hot water drilling site. (**G**) Study area location.

Here, we investigate ice shelf basal topography using an autonomous underwater vehicle (AUV) sent into the Dotson Ice Shelf (DIS) cavity in the Amundsen Sea, West Antarctica. We used an upward-looking multibeam sonar to obtain six high-resolution maps of the western, central, and eastern parts of the ice shelf base, extending up to 17 km into the cavity (Fig. 1), across an area of 140 km^2^. In addition, ocean currents, temperature, and salinity were measured 20 to 80 m below the ice. This unique dataset reveals diverse features in the ice base, including erosion patterns, flat ice plateaus bounded by steeper walls (terraces), enhanced melt in basal fractures, and previously unknown, 20- to 300-m-long, teardrop-shaped features carved upward in high-melt portions of the ice shelf. We demonstrate that the differences in basal topography can be explained by diverse basal melt mechanisms controlled largely by ocean current speed, heat content, and interaction with basal fractures.

### Dotson Ice Shelf

DIS has thinned in recent decades, largely due to excess basal melt (*10*, *28*, *32*–*34*), yet ice flow velocities and calving fluxes have remained stable or decreased (*33*). Basal melting is thought to be driven by relatively warm, salty, modified Circumpolar Deep Water (mCDW) that flows into the eastern part of the ice shelf cavity along the flank of a deep trough (*35*, *36*). This and the central section are characterized by relatively thick ice (300 to 400 m) and low basal melt rates of ~1 m year^−1^ (*10*, *34*). In the west, a colder, fresher, and shallower outflow heads northward (*37*). The large-scale geostrophic circulation (*38*) guides this relatively fast current, forming channel-like features (*10*, *28*, *34*) in the west where mean basal melt rates are ~15 m year^−1^ and the ice shelf is relatively thin (~250 m).

Current understanding of DIS topographic features comes primarily from remote sensing of the ice shelf surface. High-resolution WorldView Digital Elevation Models (Fig. 1, C to F) reveal generally thinner ice in the west compared with the east. Long, ice-front-parallel fractures are observed in the east and center, while ice velocity shear and interactions with pinning points form shorter, oblique fractures in the west. Basal channels—approximately flow-aligned troughs—are found across the ice shelf. In the east, these features are subtle and may be inherited from grounding-line topography and/or related to basal channelization of ocean water flow. In the west, they are larger, and three prominent depressions (Fig. 1B) correspond to areas of high basal melt attributed to channelized basal water flow (*10*, *28*, *34*).

### High-resolution observations of the ice shelf base

**Fig. 2.**
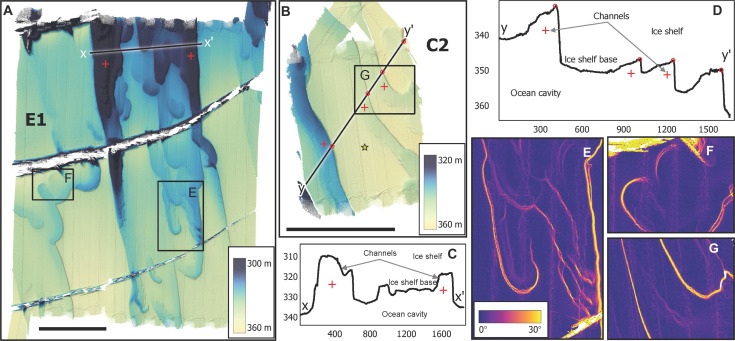
Upward-looking multibeam sonar maps of the underside of DIS. (**A** and **B**) One-meter multibeam grids showing ice base topography in the East region (E1 and C2). Color bars show the depth of the ice base. In (B), red crosses depict locations of channel-like features, and yellow star shows the location of the hot water drilling site. Profiles of the ice shelf base illustrate (**C**) channels and (**D**) terraces, with distance and depth in meters, which are also highlighted by slope derivations of the (**E** to **G**) ice base surface. Black bars in (A) and (B) are 1 km. Maps are projected in Universal Transverse Mercator zone 13S (WGS84 Datum).

**Fig. 3.**
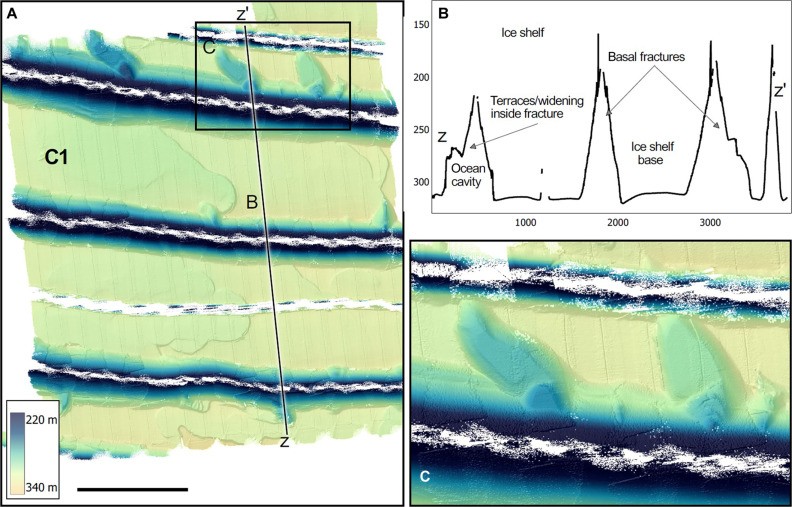
Upward-looking multibeam sonar maps of the underside of the central part of DIS. (**A**) One-meter multibeam grid from the central survey region (C1) showing full-thickness ice fractures, superposed on smaller terraces. The profile in (B) shows that the northernmost fractures are generally larger and have been widened and eroded at these flanks; themselves containing (**B** and **C**) smaller subterraces. Black bar in (A) is 1 km, and all maps projected in Universal Transverse Mercator zone 13S (WGS84 Datum).

**Fig. 4.**
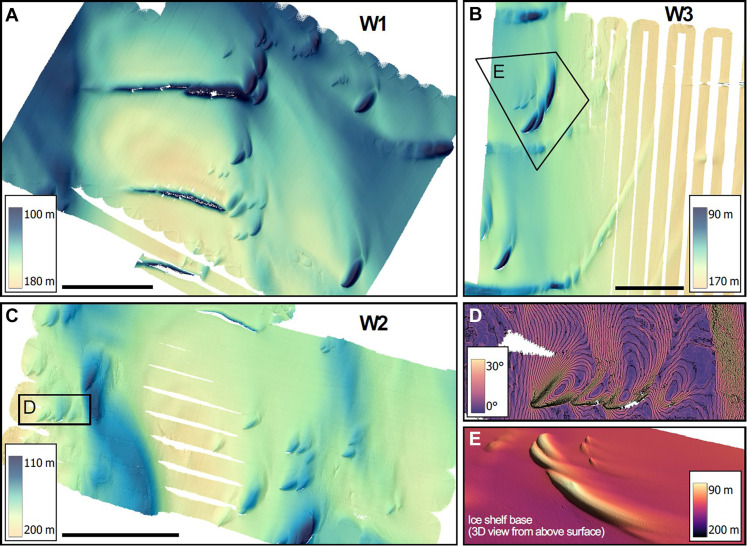
Upward-looking multibeam sonar maps of the underside of the western part of DIS. (**A** to **C**) One-meter multibeam maps from the Western region (W1 to W3) show a smooth and eroded ice topography, (**D** and **E**) enigmatic teardrops in clusters, and fractures. The 45° deviation from water flow of the teardrops is highlighted by the (D) ice base slope, and their morphology is shown in three-dimensional (3D) from a viewpoint above the (E) ice shelf base. Black bars in (A) to (C) are 1 km, and maps are projected in UTM zone 13S (WGS84 Datum).

During January to March 2022, an expedition was undertaken to investigate DIS, including several missions into the ice shelf cavity with a Hugin AUV (Fig. 1 and Materials and Methods). Six areas of the ice base were mapped in detail by an EM2040 multibeam sonar, mounted looking upward on the AUV. While all ice shelf surface features (Fig. 1) correspond to basal features in the AUV multibeam surveys (Figs. 2 to 4 and Materials and Methods), the high-resolution maps also reveal a highly complex ice base topography, including never-before-seen types of ice base formations not reflected on the surface.

The eastern and central regions (Figs. 2 and 3) are characterized by flat terraces, 200 to 2000 m wide, bounded by steep “walls” or faces, 0.5 to 5 m high, with 10° to 60° slopes. The faces form swirling patterns (Fig. 2, A, B, and E to G), and delineate areas where slabs of ice have been removed from the base. The curvature of individual steep faces resembles their closest neighbors in many regions (Fig. 2, A and E to G, and fig. S1), indicating a spatially coherent formation process. In some places, the terraces carve several levels into the ice. In region E1 (Fig. 2A and fig. S1), 5 to 10 terraces combine into two 5- to 40-m-deep patterns, aligned parallel to ice flow and bathymetric depth contours (Fig. 1). In region C1 (Fig. 3), terraces form within oblique indentations connected to large ice fractures, and in region C2 (Fig. 2B), the terraces form meandering channel-like structures in the ice base. While the average ice base is at the depth expected from hydrostatic equilibrium, the smaller-scale features deviate notably from this (e.g., compare Fig. 1E with Fig. 2B). The larger terraces often have a gently concave-up base, which might be due to bridging stresses from adjacent thin ice, which tends to hydrostatically adjust upward, deflecting the terrace’s thicker ice upward, as well.

In contrast, the base of the western (outflow) region is smooth, with shallow incised features (Fig. 4), but terraces are largely absent. The data also reveal several teardrop-shaped divots here, with an average width of 68 m (ranging from 20 to 170 m) and a height of 14 m (ranging from 2 to 50 m), based on the 75 most prominent examples. The teardrops are noticeably self-similar, with a deep indentation making a ~45° angle to the main flow at the sharp end, fanning out in a shallower and smoother rounded indentation (Fig. 4, D and E). Neither the terraces nor teardrops are visible on the surface (Fig. 1), as expected when bridging stresses prevent full relaxation to hydrostatic equilibrium (*39*).

**Fig. 5.**
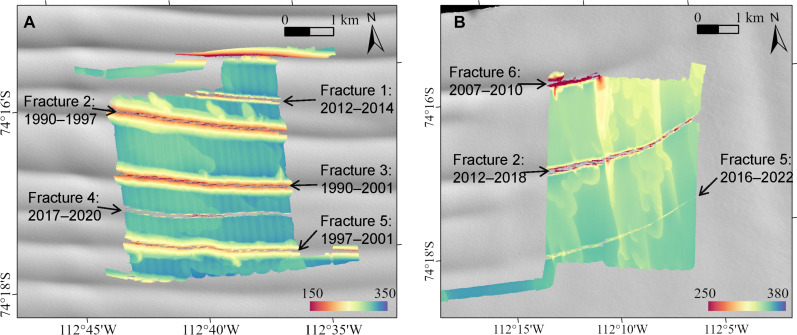
Fracture age. Approximate fracture age in the (**A**) C1 survey area and (**B**) E1 survey area. Dates show the approximate time period of fracture propagation through the ice that advected into the survey region, based on visible surface expression in Landsat imagery. Background is a Landsat-8 image from 15 February 2022.

Several large full-thickness fractures are observed, meaning that they are also detectable from above (e.g., Figs. 1D and 3A and Materials and Methods). Many of the fractures show evidence of modification by melting, such as eroded fracture bases (Fig. 3B) and indentations oblique to and contiguous with the fractures (Fig. 3C). Fracture digitization in a Landsat time series (Materials and Methods) shows that the oldest fractures in survey region C1 became visible on the surface in the 1990s, while the youngest fractures are about 2 to 5 years old (Fig. 5). The older fractures are wider and exhibit a higher concentration of contiguous melt features (Figs. 3 and 5), suggesting progressive erosion by basal melt over decades.

### Oceanographic observations

**Fig. 6.**
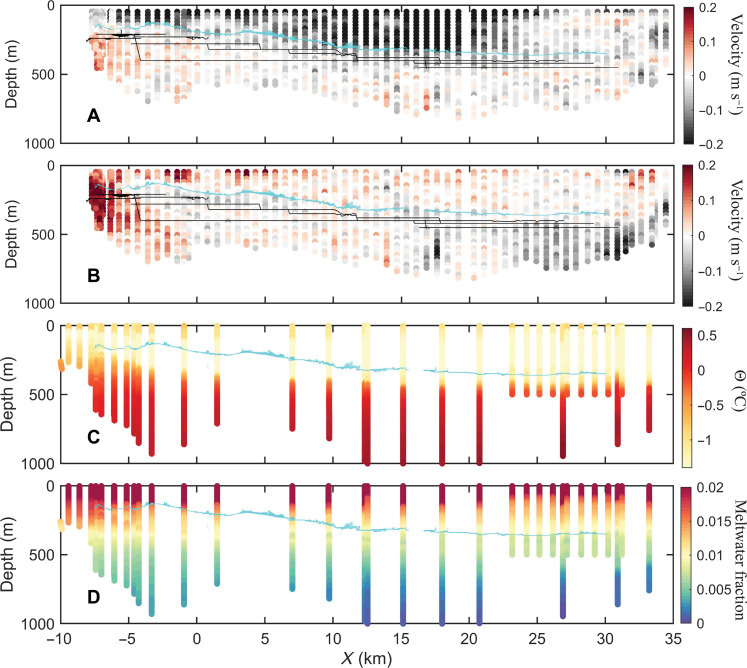
Velocity, temperature, and meltwater content collected from the ship (Materials and Methods). The viewpoint is looking north, and the distance on the *x* axis is along the ice front. Thin black lines show the depth at which the AUV moved, and cyan markers indicate the ice shelf base as measured by the AUV multibeam about 10 km into the cavity. (**A**) Eastward velocity component (m s^−1^), with negative values indicating flow toward west. (**B**) Northward velocity component (m s^−1^), with positive values indicating flow toward north. (**C**) Conservative temperature (°C). (**D**) Meltwater fraction (MWF).

In addition to the upward-looking multibeam surveys, observations of current velocities, temperature, and salinity were collected near the ice shelf front from the ship, from the AUV, and from sensors deployed through a borehole in the ice (yellow star in Figs. 1 and 2B). Meltwater concentration was calculated from temperature and salinity (Materials and Methods). Consistent with previous studies (*36*, *37*, *40*), we observe a warm, salty inflow at depth in the east, a buoyant meltwater-enriched outflow in the west (Fig. 6B), and a coastal current along the ice shelf front (Fig. 6) (*41*). Conservation of potential vorticity controlled by Earth’s rotation, local bathymetry, and ice thickness governs the large-scale exchange across the ice front (*38*).

**Fig. 7.**
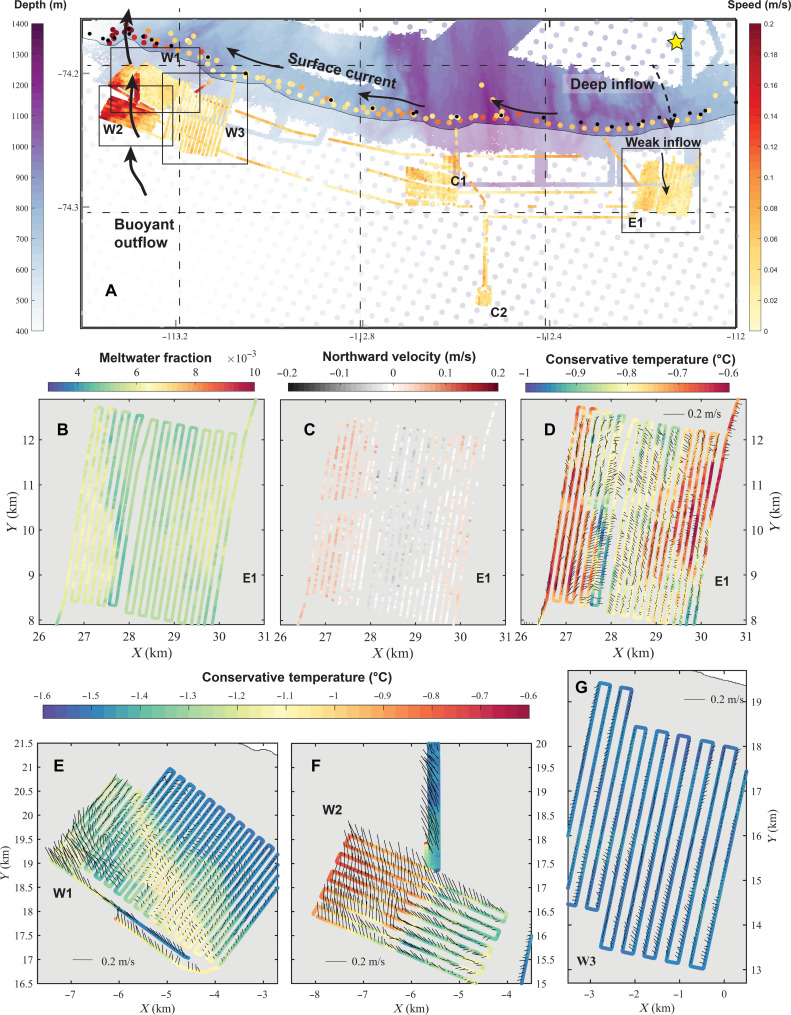
Velocity and temperature of the water inside and outside DIS cavity. (**A**) Map showing current speed in m s^−1^ according to the right-hand color bar, with black arrows showing the main currents inferred from our data (see also Fig. 6). White semitransparent area indicates the ice shelf extent, and blue shading and dots (left hand color bar) indicates bathymetry from the present cruise (Materials and Methods) and from gravity inversion (*62*). Markers inside the ice shelf are AUV data from 20 to 80 m below the ice base (Materials and Methods), markers outside the ice shelf are current speed measurements made from the ship (*61*) at the level of the ice shelf base (Materials and Methods), and black markers show the location of the ship temperature and salinity profiles. The yellow star in the top right corner shows the location of the mooring (Materials and Methods). Black squares indicate the survey areas in (B) to (G). (**B**) MWF in survey area E1 and (**C**) the north-south component of the velocity (positive northward) according to the color bar. (**D** to **G**) Velocity arrows (black) together with temperature measured by the AUV (color bars). Note that the maps in (B) to (G) show distance (km) east and north instead of latitude and longitude and that the color scale in (D) is different from (E) to (G).

Figure 7 shows the current velocity and temperature measured by the AUV below the ice base (Materials and Methods). Substantial spatial variability in temperature and meltwater content coincides with changes in velocity directions (Fig. 7, B to G, and figs. S2 and S3). Water from different sources form streaks of temperature and meltwater concentration along streamlines. The current below the central and eastern parts of the ice shelf is comparatively slow, only 0.01 to 0.04 m/s (faster flow deeper down; Fig. 6). In the east (survey region E1; Fig. 7, B to D, and fig. S3G), there is a southward flow of cold, meltwater-poor water in the center near the ice base, parallel to the basal channels and local bathymetry (Fig. 1). Survey region C1 (figs. S2A and S3E) hosts eastward flow, with a southward intrusion of colder water in the northeast corner. In the western survey regions (W1 to W3), there is a fast, meltwater-rich outflow with speeds up to 0.25 m s^−1^ (Fig. 7, E to G, and fig. S3), observed at distances 20, 50, and about 80 to 100 m from the ice base (fig. S7). Although this outflow has not before been observed inside the cavity, its location and meltwater concentration are consistent with previous studies outside the ice front (*37*, *40*, *42*).

**Fig. 8.**
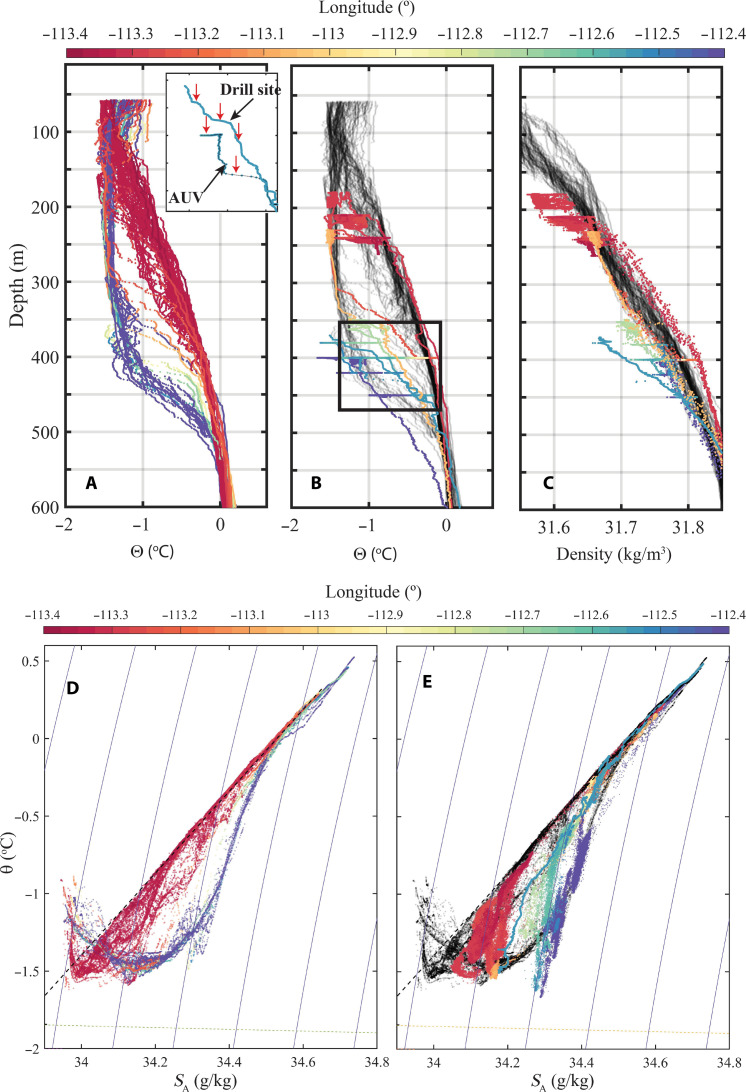
Water mass properties and basal topographic features inside the cavity. (**A**) Conservative temperature as a function of depth outside the ice shelf front, color coded by longitude (color bar). (**B**) Conservative temperature as a function of depth inside the cavity, color coded by longitude (color bar), and with pale gray markers repeating the data in (A). Inset shows profiles of conservative temperature from the borehole site, collected by the AUV, and the borehole profile, colored coded by longitude, for the parameter space indicated by the black rectangle in (B). Red arrows in inset point to the larger vertical gradients in the stairstep structures. (**C**) Density as a function of depth for the data outside the cavity (black semitransparent) and for the AUV and borehole data inside the cavity, color coded by longitude (color bar). (**D** and **E**) Temperature-salinity plots of data (D) outside the cavity and (E) inside the cavity. Color indicates longitude (color bar). Black dashed line is the meltwater mixing line, and the yellow dashed line is the freezing point. In (E), black semitransparent markers repeat the data from (D).

Remnant winter water (WW) with temperature ~−1.5°C occupies the top 350 to 450 m outside the cavity and is also found inside the cavity (Figs. 6C and 8). The outflow water, which is derived from mCDW with the addition of fresh meltwater, is up to 1°C warmer. Meltwater concentration is lowest in the east, where the ice is thickest, and increases toward the western and inner parts of the cavity (Fig. 8 and fig. S3), with well-defined melt-enriched warm streaks (Fig. 7 and fig. S3). The portion of the water column that contains mCDW mixtures increases from the eastern inflow (>500 m depth) to the western outflow (full depth) (Fig. 8). During the missions, the AUV spent long periods of time at constant depths (fig. S7), and while it is not unexpected that the density at a certain pressure is nearly constant, the great range of temperatures and (compensating) salinities encountered is an indication of water masses from different sources that interleave and find their level of neutral buoyancy inside the cavity.

## RESULTS AND DISCUSSION

**Fig. 9.**
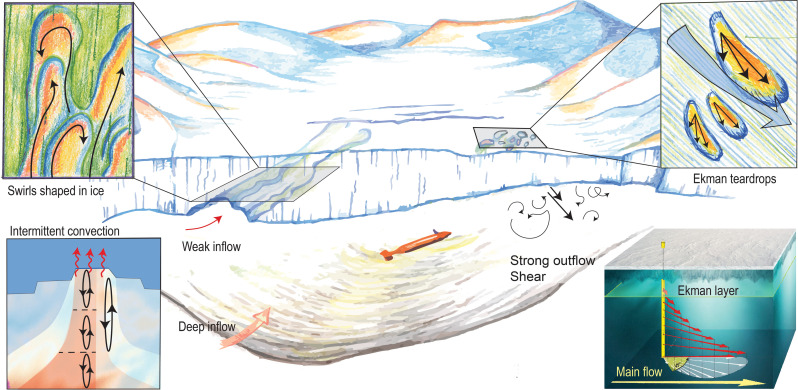
Sketch showing the processes discussed in the paper. Note that the vertical scale is exaggerated.

The observed east-to-west contrasts in basal topographic features correspond to oceanographic contrasts (Fig. 9). In the west, strong currents near the ice base give rise to a relatively smooth basal topography and high basal melt rates of 15 m year^−1^ (*28*, *34*). These are consistent with calculations using a shear-driven melt parameterization (*14*) based on our oceanographic observations (Materials and Methods). Current directions (Fig. 7, E to G, and fig. S3) align with many of the erosional features in the west (Fig. 4), which supports our hypothesis that the flowing water is creating these features. Most of the velocity data were collected between 20 and 80 m below the ice base, i.e., outside the boundary layer, but overlapping missions at varying depths generally reveal a gradient toward higher velocity and meltwater content closer to the ice here (fig. S4). This observation and the correspondence of observed melt rates with the shear-driven melt parameterization suggest that shear-driven mixing within the ice-ocean boundary layer and the presence of warm mCDW mixtures in the upper part of the water column are responsible for the high melt rates in the western outflow region.

The teardrop-shaped features (Fig. 4) are only present in the western, high-velocity region. We propose a formation hypothesis relying on Ekman theory. Near the ice-ocean boundary, frictional and Coriolis forces give rise to an Ekman spiral, similar to the benthic Ekman spiral (*9*, *43*) (Materials and Methods), developing in the 5- to 15-m-thick Ekman layer. A local disturbance in the ice base, such as a fracture tip, or large englacial rocks melting out, may initiate a turbulent plume and locally elevated melt rates. Because of the Ekman layer, such a plume is expected to spread asymmetrically with a component toward the right (in the Southern Hemisphere). The clockwise deviation from symmetry is at its maximum near the source and decreases downstream as the plume grows, spreading into the water below the ice (Fig. 9 and Materials and Methods). The observed shapes, with the tip at 45° angle to the main flow and the rounded tail fanning out, becoming more parallel to the main flow with distance from the tip, support this hypothesis. It can also be noted that the longer teardrop shapes are deeper and presumably older, with their tails more parallel to the flow, as expected. Repeat observations of the features a few months apart and/or observations of the flow velocity and turbulent heat flux inside the teardrops would be needed for definitive confirmation.

In contrast to the west, oceanographic data from central and eastern survey regions (C1, C2, and E1) show comparatively low current speeds and meltwater concentrations. The vertical profiles of temperature and salinity inside the cavity (borehole and AUV data; see Materials and Methods) show staircase-like structures in temperature Ɵ and salinity *S*_A_ (Fig. 8A, red arrows) and deviation of *d*Ɵ/*dS*_A_ slopes from the meltwater mixing line (Fig. 8), indicating that eddy diffusivities of heat and salt are unequal. Together with the Turner angle *Tu* (*44*) being −90° < *Tu* < −45° (Materials and Methods), this indicates that the water column is susceptible to convection and differential diffusion (*17*, *20*, *21*, *45*–*47*). Given the observed horizontal variations in Ɵ and *S*_A_, interleaving of water masses may play a role in producing the staircase-like features, and convection and double diffusion may reinforce them (*48*). The weakly manifested staircase steps indicate past convection rather than an ongoing process (*13*, *16*, *45*), but convection provides an avenue to get heat to the ice base when vertical shear is weak.

The ice shelf base in the central and eastern regions is deeper and comparatively level, except for pervasive rounded or elongated patterns melted into the ice. Their boundaries are sharp, forming terraced features notably different from the smooth, eroded ice of the western region. We suggest the observed terraces in area E1 form by episodic southward intrusions of warm surface water from the ice front, a process that has been observed to cause melting at Ross Ice Shelf (*7*) and is consistent with currents at the time of our survey (Fig. 7).

A mooring placed 3 to 4 km from the ice front in 2014–2016 (figs. S5 and S6) documented episodic southward intrusions of warm water at the level of the ice base (*36*), strong enough to cause the observed terraces. A more likely source for the channel-like features in C2 further south is warm and buoyant CDW-meltwater mixtures from the inner cavity moving out.

The sharp delineation of the melt features points to ocean processes that are stable for long enough to be imprinted on the ice base and the existence of a reinforcing melt topography feedback. In survey region E1, several elongated terraces are superposed on one another, aligned in the north-south direction, and overlapping with the outer part of the long basal channels originating near the grounding zone (dashed lines, Fig. 1). This indicates that the intrusions may be topographically steered by the basal channels and that the melt features themselves can guide further inflows. This is a similar process to seabed topographic steering (*49*), whereby intrusions of water near the ice base are redirected to follow previous paths. Melt topography feedback would explain why several terraces are superposed in the same location (Fig. 2 and fig. S1), as well as their well-defined edges. Two other mechanisms have been suggested for terrace formation: forcing from buoyant plumes, the speed of which is set by the ice slope (*14*, *23*), or control of melt rates by variable stratification across basal slopes (*9*, *23*, *25*). Neither of these can solely explain the observed features, although they might contribute and could be dominant in other parts of the ice shelf. To definitively determine how the terraces form, repeat surveys showing their evolution, preferably combined with observations from the boundary layer during their formation, would be needed.

The eastern and central regions are characterized by the presence of full-thickness fractures. The older fractures show considerable basal melt and widening compared with younger ones. Elevated water velocities within fractures have been observed below Thwaites Ice Shelf (*25*) and Kamb Ice Stream (*30*, *31*), which can explain the enhanced melting. However, it is also possible that the relatively warm and buoyant meltwater mixtures formed in the cavity (Fig. 8) accumulate in the fractures. The oblique protuberances at older fractures (Fig. 3) have not been observed before, and their origin is unknown. They may be caused by locally stronger currents, modified by rotation, and/or episodic flushing of warmer water from inside the fracture. These imprints, together with recent work on flow near fractures (*25*, *30*, *50*), show that current parameterizations of basal melt, a key component of ice shelf stability (*26*, *51*), are missing many physical processes. Fractures in the western region do not display such features, suggesting that the high basal melt in a shear-dominated regime outpaces their formation.

The fractures may also place age constraints on the terraces. The youngest fracture in E1 (the southern fracture; formed 2016–2022; Fig. 5 and Materials and Methods) splits the terraces cleanly, indicating that those terraces formed before the fracture, while the two older fractures (formed 2007–2018) appear to have influenced the formation of terraces and other melt traces (Figs. 2A and 5). Melt features are deeper immediately south of the older fractures, demonstrating higher melt rates after flow crosses the fracture. This may be due to topographically enhanced turbulence on the downstream side (*52*) or warmer water within the fracture.

Our data show a clear picture of contrasting oceanography tied to ice shelf basal topography demonstrating the spatially variable melt processes controlling basal melt on DIS. Some features, such as the Ekman-induced teardrops, and the clear contrast between the low–melt rate terraces and the high–melt rate western outflow are previously unknown. While the topographic patterns show the effect of basal melt on the ice, more information from the ice-ocean boundary during their formation is needed to fully understand the processes. Shear-driven turbulence, convection, and slope-dependent melting are likely all active to certain degrees, and in situ data from, or modeling of, the actively melting ice-ocean boundary are needed to better quantify their contribution. Wider-scale mapping of ice shelf bases, e.g., via one-off and repeat multibeam surveys, may also be used to further define and quantify critical and unknown physical ice-melt mechanisms. This can offer the key missing insights required to improve modeled projections of ice shelf melt and sea level rise.

## MATERIALS AND METHODS

### Experimental design

The AUV used in this study is a Kongsberg model Hugin, with 3000-m depth rating. The AUV is equipped with an upward-looking multibeam sonar, model EM2040 CX, that was operated at 300-kHz frequency. The initial quality control of the multibeam data was done with EIVA NaviModel software, which has a capacity to analyze upward-looking multibeam data and to filter out data of quality 13 or lower. Areas of no signal return, e.g., caused by too steep inclination angle, appear as white patches in Figs. 2 to 4. For velocity data from the AUV, the two Doppler velocity loggers were used, an upward- and a downward-looking Nortek 500 kHz. These were also used for navigation. In addition to these sensors, dual SeaBird SBE-19plusV2 conductivity-temperature-depth (CTD) systems, including two SBE43 dissolved oxygen sensors and pumped with two SBE5T pumps, were mounted and measuring at 4 Hz. Three presurvey missions near the seabed were also conducted; for these, a Pinnacle 45 acoustic doppler current profiler (ADCP) operating at 45 kHz was mounted instead of the upward-looking multibeam (fig. S8).

In total, four successful missions were conducted, surveying the base of DIS, resulting in six high-resolution maps of the ice. To achieve accurate navigation aided by bottom (or ice) track, the AUV needs to be within 100 m of a solid boundary—either the seabed below or the ice above. To minimize the time spent without bottom or ice tracking, the AUV was programmed to initially dive directly to the seabed and swim near the seabed, until it was inside the ice cavity. Once inside the cavity, it would ascend to the ice and perform surveys near the ice. After the surveys, it would dive down to the seabed, exit the cavity, and ascend to the surface for recovery. This operation is illustrated in fig. S7, which shows the principle of operation together with the vehicle depth during missions and the measured ice draft. Dives and ascents underneath the ice produced a series of profiles of velocity as well as temperature and salinity stratification.

To aid the mission planning, information was obtained from satellite imagery and from three presurvey missions with the AUV following the seabed. In the three presurvey missions, the ice draft was measured with a long-range ADCP (a Pinnacle 45 kHz), with 1000-m range. The backscatter data were used to get estimates of the ice draft, necessary for safe mission planning for the ice surveys (fig. S8). Safe areas for deployment and recovery were identified using updated positions of Dotson ice front and prominent icebergs. These were delineated using the blue bands of Landsat 8 (~0.483-μm central wavelength; 30-m spatial resolution) or Sentinel-2B (0.490 μm; 10 m) imagery taken close in time to the mission. The images were screened, and cloud-covered pixels were masked, using the respective quality-assessed bands of each satellite (qa_pixel on Landsat 8 and Scene Classification Layer on Sentinel-2), before processing. To detect the ice front, we applied the scikit-image find_contours tool (*53*), which applies a marching squares method to delineate contours (*54*). This method contoured the ice front, icebergs, and other features in the image. We then selected the ice front using the longest contour, visually verifying the accuracy of our choice to ensure that erroneous features were not inadvertently selected.

### Navigation and ADCP data quality control

The Kongsberg AUVs are fitted with a high-fidelity aided inertial navigation system (AINS), which also includes a sea current estimator (*55*–*57*). Similarly, a suite of postprocessing algorithms is available to further reduce uncertainties (*58*). The latter suite was used for improving the georeferenced AUV data in this article. The output from the in situ and postprocessed navigation includes position and depth, velocity, orientation, and associated uncertainties. Navigation postprocessing uses all the raw and precisely timestamped navigation sensor data acquired in-mission, including GPS before descent and after ascent, and both upward- and downward-looking ADCP/DVL. It then reprocesses the navigation solution by running optimal stochastic smoothing (*58*). Figure S9 shows the navigation before (red) and after (black) postprocessing for regions W2, W3, C1, E1, and C2 (region W1 was indistinguishable from the raw position). Last, the fractures seen in a satellite image acquired on 15 February 2022 were compared with the postprocessed map. Because of the irregular ice shelf base, there were occasions when the upward-looking DVL lost ice track, such as when it travelled below one of the many fractures. This situation gives location errors in the raw data of up to 300 m. After postprocessing, these errors were reduced.

An example of the current velocity postprocessing is shown in fig. S10 for transiting to and surveying area W2. The AUV was operated with speed control applied and hence had a stable speed in the surge (along-vehicle) direction. The spikes or transients occur during turns or other dynamics. The AUV transited close to the seafloor while later going up under the ice. The bottom track data provide the AUV’s velocity over ground (measured by the downward mounted ADCP/DVL) and, similarly, for ice track, velocity relative to the near-stationary glacier ice (measured by the upward mounted ADCP/DVL). The AUV’s velocity relative to the water masses was also measured, denoted water track.

From the bottom/ice track velocity, water track velocity, and orientation, it is possible to directly derive an estimate for ocean current (fig. S11). The raw ocean current is found by subtracting the water track data from the earth/ice track data, followed by a rotation to geographical coordinates. The ocean current time series, including the transition from transit (based on bottom track) to survey (based on ice track), show good consistency, without any large shifts when switching between the two modes. To reduce noise, the velocity data were binned in 30-s bins. It was also detided using the Cats2008 model (*59*). Together with the binned data, fig. S11 also shows the ocean current estimated by the AINS. The latter is based on a Kalman filter implementation where the sea current is modeled as a stochastic process. The binned data show good agreement with the AINS output.

### Fracture age estimation

To approximate the age of the fractures observed in the survey region, we compiled a time series of imagery from the Landsat record. The earliest available image with enough time continuity to track fracture development was acquired 23 January 1990, and we continued the time series through 15 February 2022, close to the time of the bathymetric survey. When possible, two cloud-free images per summer season were included (see table S1 for list of images), but before 2012, this was rarely possible, and only two images were available between 1990 and 2000.

In each image, five primary fractures visible in the AUV data, which corresponded closely to the locations of fractures visible in the 15 February 2022, Landsat image, were digitized as far back in the record as they were visible (two examples are shown in fig. S12). The date that a fracture becomes visible on the surface may be a while after fracture formation, as the fracture size and bridging stresses limit hydrostatic relaxation of the ice shelf plate that allows them to become visible in optical imagery. In addition, differences in lighting angle make it difficult to pinpoint fracture locations, particularly near the ends of fractures where the surface expression is less clearly defined. Despite these uncertainties, we expect overall trends to be reliable in the record, and consistent methodology allows for the comparison of relative fracture ages.

The fractures in the C1 survey region (Fig. 5A) all originated from the west of the survey area and propagated to the west. Fractures 2, 3, and 5 were visible to the west of the ice that entered the survey region in the first image in our survey, from 1990. Fracture 2 propagated between 1990 and 1997, fracture 3 between 1990 and 2001, while the bulk of the propagation in this region for fracture 5 occurred between 1997 and 2001. Fractures 1 and 4 are much younger. Fracture 1 first appeared in imagery in February of 2012 and propagated across the survey region approximately between 2012 and 2014. Fracture 4 first became visible to the west in February 2015 but did not propagate across the survey region until approximately between 2017 and 2020.

### Description of the shipborne CTD and the ship ADCP

The expedition to the DIS front region was undertaken from the RV/IB *Nathaniel B. Palmer* during January to March 2022. A SeaBird SBE 911plus CTD package with dual pumped temperature and conductivity sensors, mounted on a 24-bottle rosette, was deployed from the vessel. Both pairs of sensors were pre- and postcruise calibrated by SeaBird, and the final quality-controlled dataset includes corrections that were well within the reported accuracy of the SBE temperature and conductivity sensors (0.001°C and 0.0003 S m^−1^, respectively). The data were processed using the standard SBE Data Processing package (including cell thermal mass corrections and pressure loop flags), and the downcasts were averaged in 1-dBar bins. Absolute salinity and conservative temperature were then calculated using the TEOS-10 Gibbs-SeaWater toolbox (*60*) (https://teos-10.org/software.htm). Suspect and noisy data, primarily near the surface, were removed during quality control.

Data from the two hull-mounted ADCPs, a 38- and a 75-kHz Ocean Surveyor from Teledyne RD Instruments, both operating in narrowband mode, were also used. Data from both ship ADCPs were logged and processed using the University of Hawaii Data Acquisition System in 5-min ensembles (*61*). The ship ADCPs were pinging almost continuously through the cruise, but data quality varied with sea and ice conditions. All velocity data were detided using the CATS2008 model (*59*).

### CTD data from the AUV

The AUV is equipped with three CTD instruments: two SeaBird SBE19plusV2 pumped systems and one SAIV (mounted outside the hull). The SAIV CTD has lower resolution conductivity data, especially at higher pressures, but has the advantage of measuring in situ without pumping. Figure S13 shows how the SeaBird sensors are mounted inside the hull with the tubing extending outside the vehicle boundary layer. The raw data were processed following the SeaBird standard protocol, and offsets were identified on the basis of the postcruise calibrated ship CTD data and corrected in the final dataset. These offsets were +0.0026 S m^−1^ for conductivity and +0.0002°C for temperature. The data were also despiked and bin-averaged from 4 Hz in the raw data to 1 Hz. Figure S13 shows plots of conservative temperature versus absolute salinity of the ship CTD data together with the AUV and borehole data before and after offset adjustment.

### Borehole CTD

We make use of an exploratory CTD cast through a hot water borehole at 74° 22′ 12.53″ S, 112° 32′ 26.27″ W (74.370 S, 112.541 W). A Seabird SBE49 (SN 0219) FastCAT CTD profiler was mounted on a purpose-built deployment frame and attached to a winch cable with a mechanical termination and an electrical termination for live data collection. A downward and upward cast of temperature and salinity with pressure was made at 14:46 UTC on 7 February 2022, sampling at a rate of 16 Hz. Data collection took about an hour each way. The CTD spent a couple of minutes at the seabed (1226 dB, recorded by an RBRsolo), which introduced artifacts in the salinity observations during the upcast. The data were filtered, aligned in time, and averaged into 0.1-m vertical bins using the Seabird processing routines version 7.26.7.129. No thermal mass corrections were applied. The data were calibrated with CTD data from the RV/IB *Nathaniel B. Palmer* and CTD data from the AUV (fig. S13), and conductivity was shifted by +0.0015 S m^−1^. The manufacturer-stated accuracy of the temperature and conductivity sensors are ±0.002°C and ±0.0003 S m^−1^, respectively.

### Bathymetry

The bathymetry in Fig. 7 is obtained from (i) downward-looking multibeam from the RV/IB *Nathaniel B. Palmer*, a Kongsberg EM122 that was operated continuously through the cruise with a frequency of 12 kHz, (ii) downward-looking multibeam from the AUV, an EM2040 that was operated at 300 kHz, together with (iii) a bathymetric compilation of existing multibeam data and gravity inversions (*62*).

### Meltwater fraction

Meltwater fraction (*MWF*) is calculated using the standard source equation based on salinity and temperature (*63*, *64*)

$$\text{MWF}=\frac{\left[\theta -\theta_{\text{MCDW}}-\frac{\left(\text{SA}-{\text{SA}}_{\text{MCDW}}\right)}{\left({\text{SA}}_{\text{WW}}-{\text{SA}}_{\text{MCDW}}\right)}\left(\theta_{\text{WW}}-\theta_{\text{MCDW}}\right)\right]}{\left[\theta_{\text{MW}}-\theta_{\text{MCDW}}-\frac{\left({\text{SA}}_{\text{MW}}-{\text{SA}}_{\text{MCDW}}\right)}{\left({\text{SA}}_{\text{WW}}-{\text{SA}}_{\text{MCDW}}\right)}\left(\theta_{\text{WW}}-\theta_{\text{MCDW}}\right)\right]}$$ (1)

where θ is the conservative temperature (°C), *SA* is the absolute salinity (g/kg), and subscripts MCDW, WW, and MW denote the properties of θ and *SA* for respective water mass, with MCDW being the local mCDW, WW the winter water, and MW the meltwater (adjusted for the latent heat transfer). On the basis of the survey data and the latent heat budget (*63*, *64*), the values listed in table S2 are used.

### Estimates of shear-driven basal melt (three-equation model)

Shear-driven basal melt can be estimated using the three-equation parameterization (*14*). Using standard heat and salt transfer coefficients (*14*) and approximate values of ocean temperature and velocity observed below the ice shelf (velocity between 0.15 and 0.25 m s^−1^ and temperature between −1.2° and −1.7°C), the expected melt rates under shear-driven melting is calculated and shown in table S3. The results show that a basal melt rate of ~10 to 15 m year^−1^ is expected in the western region.

### Turner angle and stability diagram

The Turner angle (*Tu*) quantifies the relative contributions of the vertical gradients of conservative temperature and absolute salinity to the vertical stability/stratification of the water column (*44*). Tu expresses the susceptibility to double diffusion processes that might lead to vertical turbulence and mixing. The calculation was performed following TEOS-10 equations (*60*), which describes *Tu* as

$$\text{Tu}={\text{tan}}^{-1}\left(\alpha \frac{\partial \theta }{\partial z}+\beta \frac{\partial S_{A}}{\partial z},\alpha \frac{\partial \theta }{\partial z}-\beta \frac{\partial S_{A}}{\partial z}\right)$$ (2)

where α and β are, respectively, the thermal expansion and the haline contraction coefficients, and ∂θ/∂z and ∂*S*_A_/∂z are the conservative temperature and absolute salinity vertical gradients, respectively. The water column is considered stable when −45° < *Tu* < 45°. When −90° < *Tu* < −45°, diffusive convection is possible (i.e., heat diffusion dominance). Salt fingering (e.g., when relatively warm, salty water is above relatively colder, fresher water) is expected when 45° < *Tu* < 90°. A statically unstable condition is achieved when *Tu* < −90° and *Tu* > 90°. *Tu* was calculated from the vertical profiles, i.e., from the borehole CTD and the AUV dives and ascents underneath the ice (figs. S4 and S7). The dive angle for the AUV is 45°, and it was assumed that the vertical changes are small compared to horizontal during dives. Before the *Tu* calculation, θ and *S*_A_ were 10-m running mean filtered to deemphasize fine scale structure. Our calculation revealed that most of the water column in the cavity have −90° < *Tu* < −45° near the ice base (fig. S14), indicating a thermally unstable system with relatively warm waters beneath colder waters. Diffusive convection might play an important role in vertical heat flux on regimes where shear-driven flow is weak (*13*, *16*, *21*).

### Ekman layer below the ice

Like benthic boundary layers, an Ekman layer can form at the ice-ocean interface. The governing equations inside the Ekman layer are given by (*43*)

$$\begin{array}{c} \mathrm{fv}=\nu \frac{\partial^{2}u}{\partial z^{2}}\\ -\mathrm{fu}=\nu \frac{\partial^{2}v}{\partial z^{2}} \end{array}$$ (3)

where *f* (s^−1^) is the Coriolis parameter, *v* (m^2^ s^−1^) is the kinematic viscosity, *u* and *v* (m s^−1^) are the horizontal velocities, and *z* (m) is distance from the ice interface. Eliminating *v* from Eq. 3 gives the following fourth-degree differential equation

$$\frac{\partial^{4}u}{\partial z^{4}}+\frac{f^{2}}{\nu^{2}}u=0$$ (4)

Using the following four boundary conditions

$$\begin{array}{c} u\left(0\right)=0\\ v\left(0\right)=0\\ \underset{z\to -\infty }{\text{lim}}u\left(z\right)=0\\ \underset{z\to -\infty }{\text{lim}}v\left(z\right)=v_{G} \end{array}$$

where *v*_G_ is the velocity in the interior, gives the solution

$$\begin{array}{c} u=-v_{G}e^{\frac{z}{\delta_{E}}}\text{sin}\left(\frac{z}{\delta_{E}}\right)\\ v=v_{G}\left(1-e^{\frac{z}{\delta_{E}}}\right)\text{cos}\left(\frac{z}{\delta_{E}}\right) \end{array}$$ (5)

to Eq. 4, where the Ekman layer thickness δ_E_ is given by

$$\delta_{E}=\sqrt{\frac{\upsilon }{2f}}$$ (6)

Using a quadratic drag law (*65*), δ_E_ can be estimated to 5 m following

$$\delta_{E}=\sqrt{\frac{\upsilon }{2f}}\approx \frac{C_{D}|v_{G}|}{f}\approx 5\;m$$ (7)

where *C*_D_ = 3·10^−3^ is the quadratic drag coefficient, *v*_G_ = 0.25 m s^−1^ (from the velocity data in Fig. 7), and *f* = 1.4·10^−4^ s^−1^ is the Coriolis parameter at DIS. Equation 7 is the dynamical equivalent to the Ekman layer thickness when using a quadratic drag law (*65*). Equation 5 is shown in fig. S15. As the velocity is forced to zero at the solid boundary, it also simultaneously veers to the right of the main flow direction (in the Southern Hemisphere). At the boundary, the angle is 45° to the right of the main flow, i.e., northeast if the main current is northward.

Because of the veering of the Ekman layer, a source for turbulence and/or heat originating at the ice surface will not spread symmetrically in the direction of the main velocity according to, e.g., Gaussian plume dynamics. Instead, it will initially spread 45° to the main flow near the source, and then more and more parallel to the main flow as the plume grows, incorporating more and more of the interior. We hypothesize that a turbulent plume initiated at the ice-ocean interface can cause the teardrop-shaped ice features observed in the western region to melt out in this manner.

### Excursion and melt pattern thickness based on mooring data

The energy *E*_M_ (J) required to melt a volume of ice is given by

$$E_{M}=L\rho_{\text{ICE}}V_{\text{ICE}}$$ (8)

where *V*_ICE_ is the ice volume (m^3^), ρ_ICE_ is the ice density (kg m^−3^), and *L* (J kg^−1^) is the latent heat of fusion for ice. The energy *E*_C_ (J) that can be supplied by a convecting water volume below the ice is given by

$$E_{C}=C_{P}\Delta T\rho_{W}V_{W}$$ (9)

where *C*_P_ (J kg^−1^ K^−1^) is the specific heat capacity of water, ρ_W_ (kg m^−3^) is the density of water, Δ*T *(K) is the decrease in water temperature during convection, and *V*_W_ (m^3^) is the volume of the convecting layer. Assuming that water flows into the cavity with speed *U* (m s^−1^), the incursion length *I*_W_ (m) is given by

$$I_{W}\leq \mathrm{Udt}$$ (10)

During convection, the maximum energy flux *F*_M_ (W) from such an inflow to the ice base is given by

$$F_{M}=U\rho_{W}{\mathrm{BH}}_{W}C_{P}\left(T-T_{F}\right)$$ (11)

where *U* (m s^−1^) is the velocity of the inflow, *B* (m) is the width of the inflow, *H*_W_ is the thickness of the convection layer, *T* is the average temperature of the convection layer, and *T*_F_ is the freezing point. Using Eqs. 8 and 9, and assuming that the outline of the melt patterns is the same as the outline of the area occupied by the convecting fluid, gives a relation between the thickness *H*_ICE_ of the melt patterns and the thickness of the convecting layer

$$H_{\text{ICE}}=C_{P}\left(T-T_{F}\right)\frac{H_{W}}{L}$$ (12)

Figure S5 shows 2 years of data from near the ice front (*36*) on the eastern side (yellow star in Fig. 7A), used to calculate the maximum excursion (Eq. 10) and the ice melt (Eq. 12) in fig. S6. For the excursion length (Eq. 10), the average velocity between 400 and 250 m depth was bin-averaged to 12 hours, and then *I*_W_ was calculated using dt = 12 hours. For the thickness of the ice melt (Eq. 12), the temperature *T* was the average temperature between the ice base and the assumed thickness of the convecting cell (*H*_W_) and similarly bin-averaged every 12 hours. Three different *H*_W_ were used: 50, 100, and 150 m.

## Supplementary Materials

### This PDF file includes:

Figs. S1 to S15 Tables S1 to S3 References
