# Supplementary material for: Swirls and scoops: Ice base melt revealed by multibeam imagery of an Antarctic ice shelf
Source: Sci Adv. 2024 Jul 31;10(31):eadn9188. doi: 10.1126/sciadv.adn9188 (PMC11290488; doi:10.1126/sciadv.adn9188)
Supplement: Supplementary file 1 — Figs. S1 to S15 Tables S1 to S3 References [file sciadv.adn9188_sm.pdf]

Science Advances
AAAS

Supplementary Materials for

Swirls and scoops: Ice base melt revealed by multibeam imagery of an Antarctic ice shelf

Anna Wåhlin *et al.*

Corresponding author: Anna Wåhlin, anna.wahlin@gu.se

Sci. Adv. **10**, eadn9188 (2024)
DOI: 10.1126/sciadv.adn9188

This PDF file includes:

Figs. S1 to S15
Tables S1 to S3
References

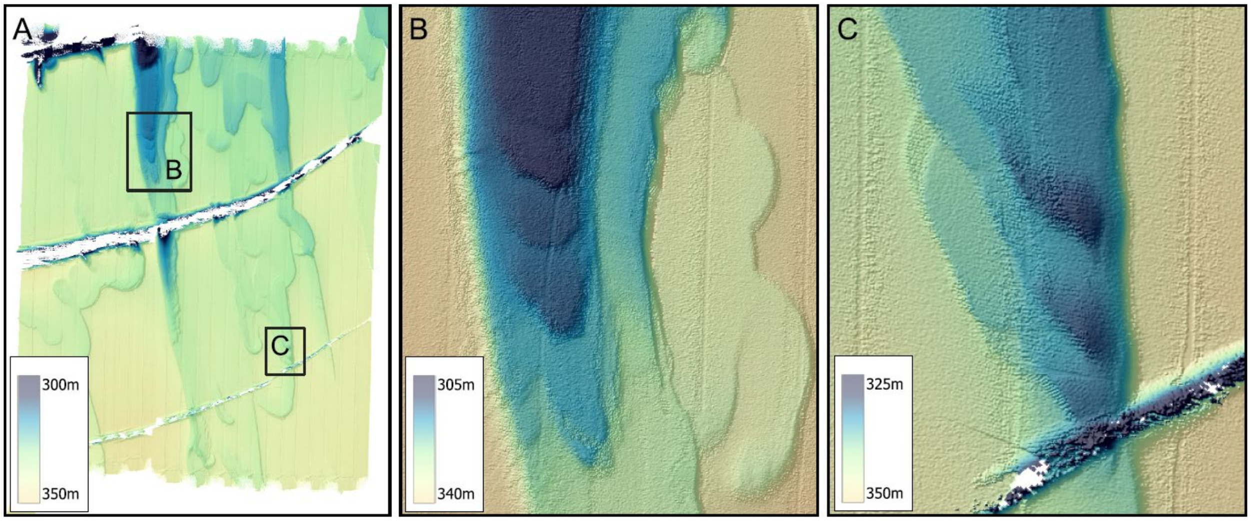

Figure S1. Upward-looking multibeam sonar maps of the underside of the eastern part of Dotson Ice Shelf. (A) 1-m multibeam grid from the eastern survey region (E1). Black squares in (A) show the zoomed-in areas in (B) and (C) where melt patterns are superposed on each other. All maps projected in UTM Zone 13S (WGS84 Datum), ice draft is given by color bars in each panel.

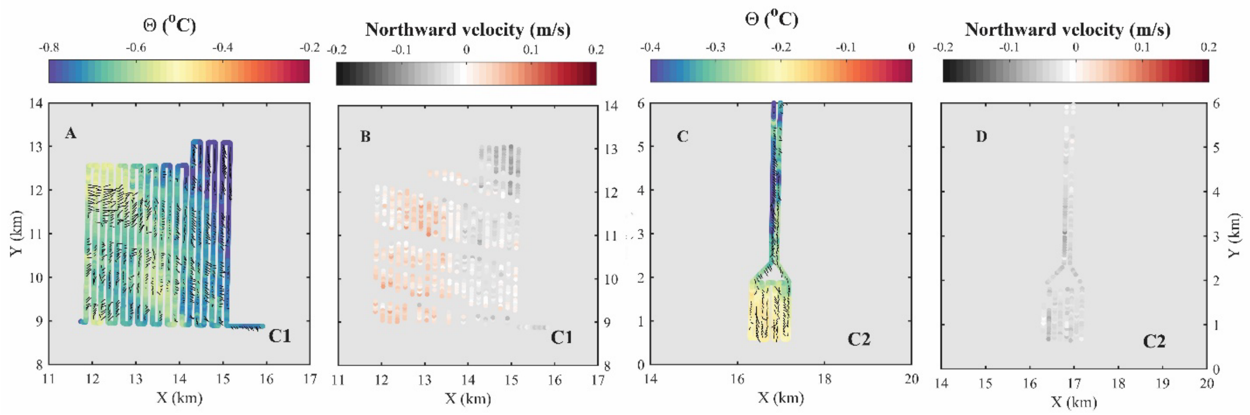

Figure S2. Velocity and temperature for survey areas C1 and C2. (A) arrows (black) together with Conservative Temperature Θ (°C) measured by the Autonomous Underwater Vehicle (AUV) (color bar) for survey area C1 (B) Northward velocity component (color bar) for survey area C1 (C) arrows (black) together with temperature measured by the AUV (color bar) for survey area C2 (D) Northward velocity component (color bar) for survey area C2

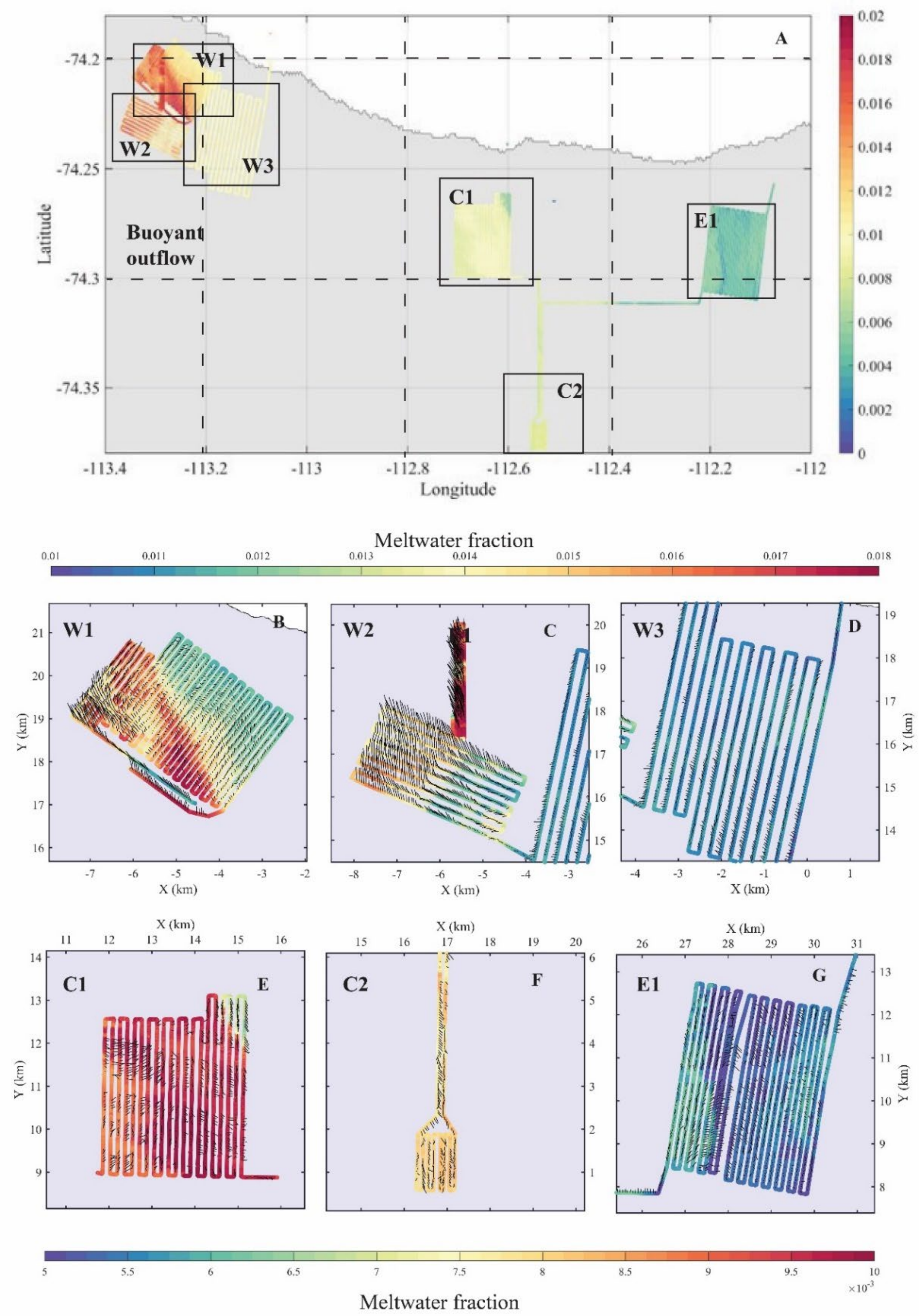

Figure S3. Meltwater inside the cavity is concentrated to the western side and northward currents. (A) Map showing the meltwater fraction (color bar) along the Autonomous Underwater Vehicle (AUV) mission paths. Panels B-G shows the survey regions overlaid with velocity vectors (same as in Fig. 5 in main paper). Note that the color bar in (B) - (D) is different from the one in (E) -(G).

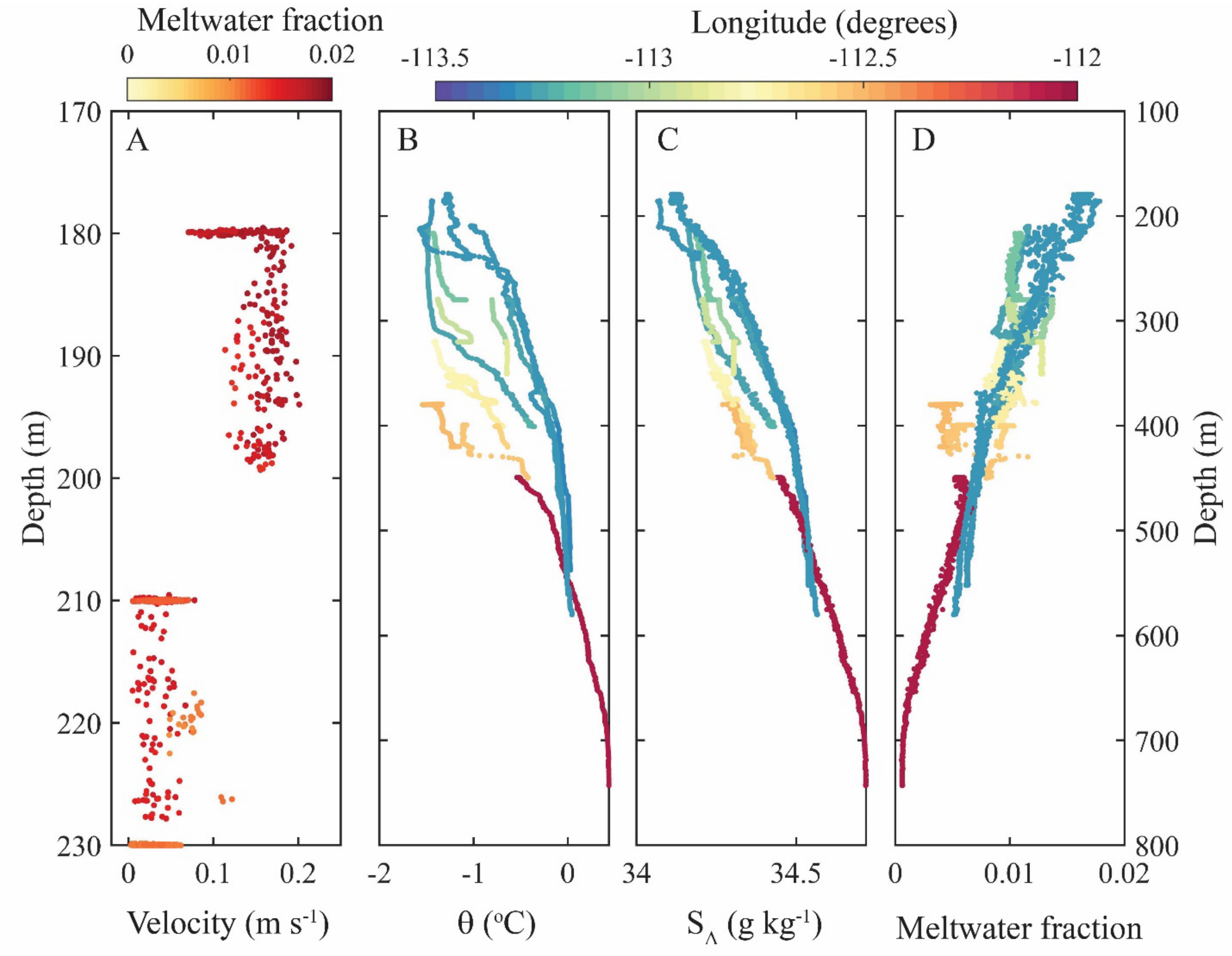

Figure S4: Vertical profiles of water properties inside the cavity. (A) Vertical profile of velocity, color coded by meltwater fraction (color bar) for the overlapping lines (mission 9, deeper data and mission 14, shallower data) in survey area W1. Panels (B) - (D) show vertical profiles, from the dives and ascents underneath the ice (Fig. S7), of (B) conservative temperature, (C) Absolute salinity, and (D) meltwater fraction, color coded by longitude.

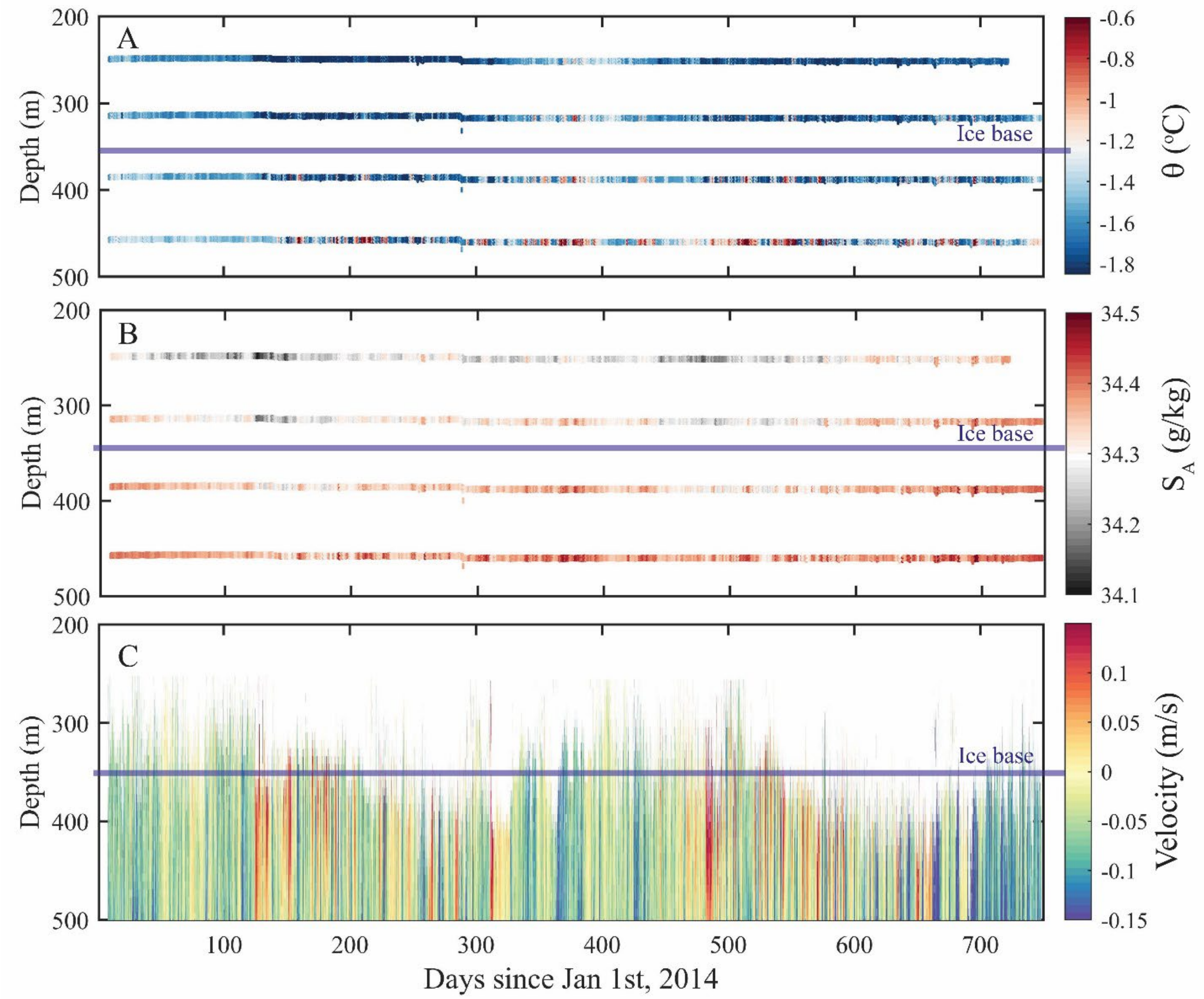

Figure S5. Mooring data from the eastern cavity front region. Data from a mooring placed near the ice front (for location see Fig. 2b) during 2014 and 2015. (A) Hourly conservative temperature Θ (color bar) (B) Hourly absolute salinity S_A (color bar) (C) North-south velocity component (northward positive)

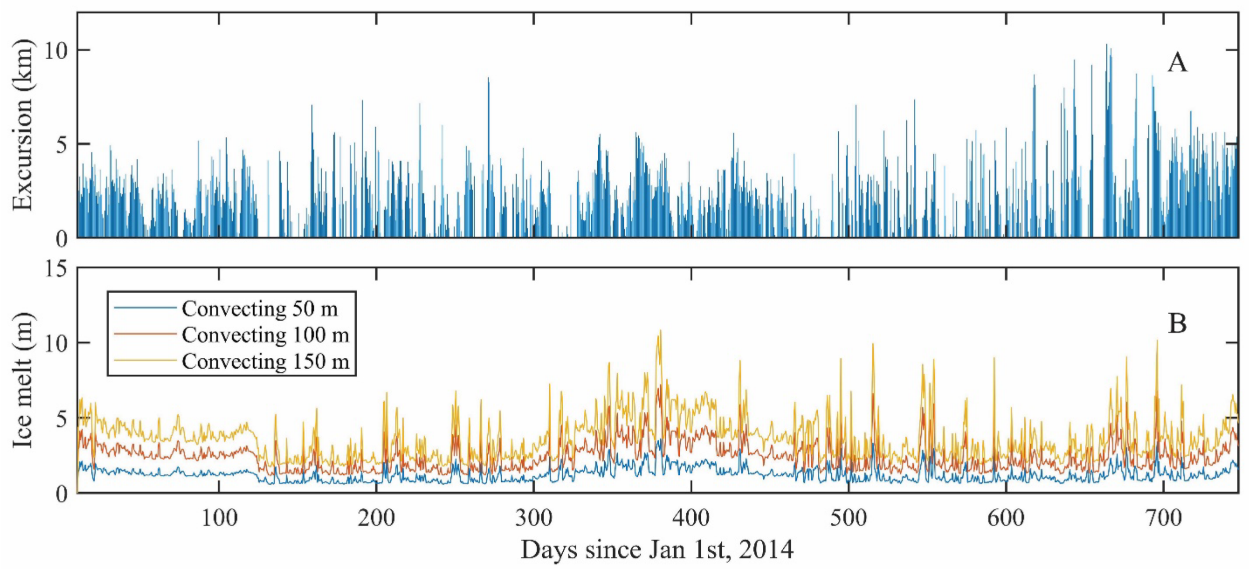

Figure S6: Statistics of excursion and melt from mooring data. (A) Maximum excursion of warm water below the ice shelf (km) based on mooring data (methods) (B) Ice melt (m) based on mooring data (methods)

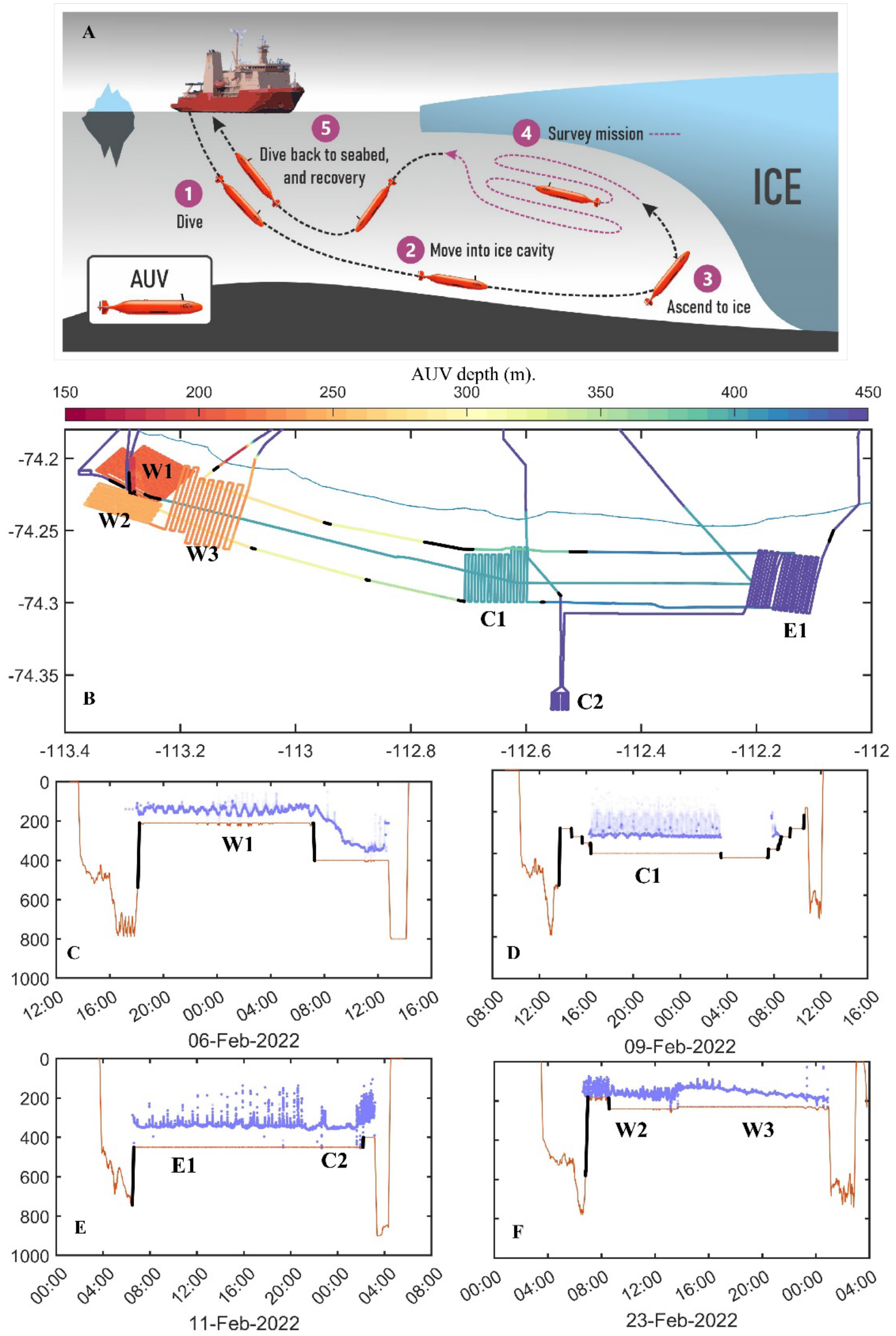

Figure S7: Overview of the Autonomous Underwater Vehicle (AUV) mission design. (A) Sketch showing the different mission phases (B) Map showing the AUV depth for all missions, with black markers indicating the dives and ascents, survey names indicated. (C) - (F) Time-depth profiles showing the AUV depth (thin orange line) as a function of time, with black lines indicating the dives and ascents inside the cavity. Semi-transparent blue markers indicate the ice draft obtained from the upward-looking multibeam sonar.

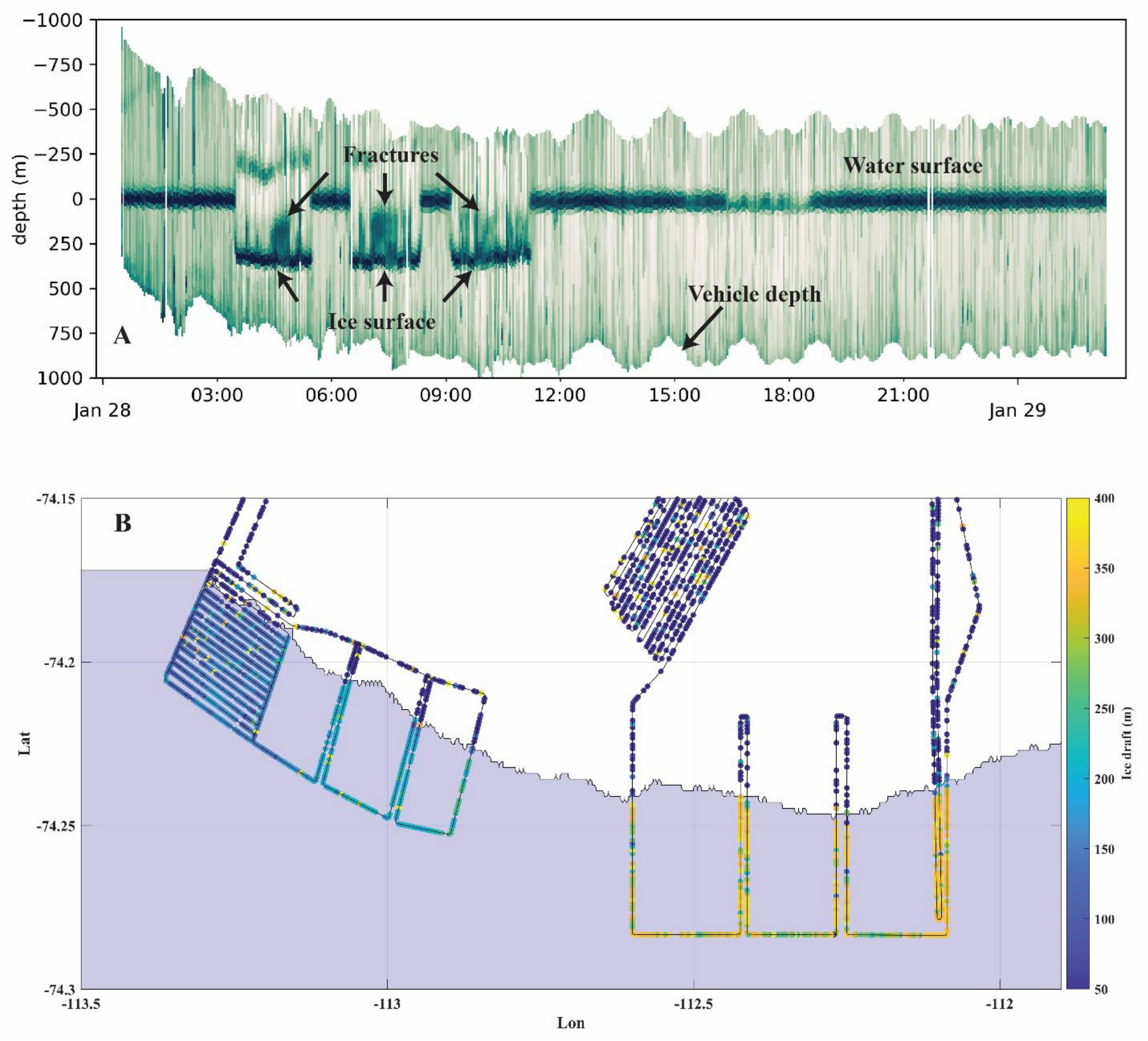

Figure S8. Data from the long-range Acoustic Doppler Current Profiler (ADCP) during the three pre-survey missions. (A) Backscatter intensity as a function of time and depth in pre-survey mission 3, with arrows marking water surface, ice surface, and suspected fractures. (B) Overview map of the ice draft (defined as the level of maximum intensity) from all three pre-survey missions

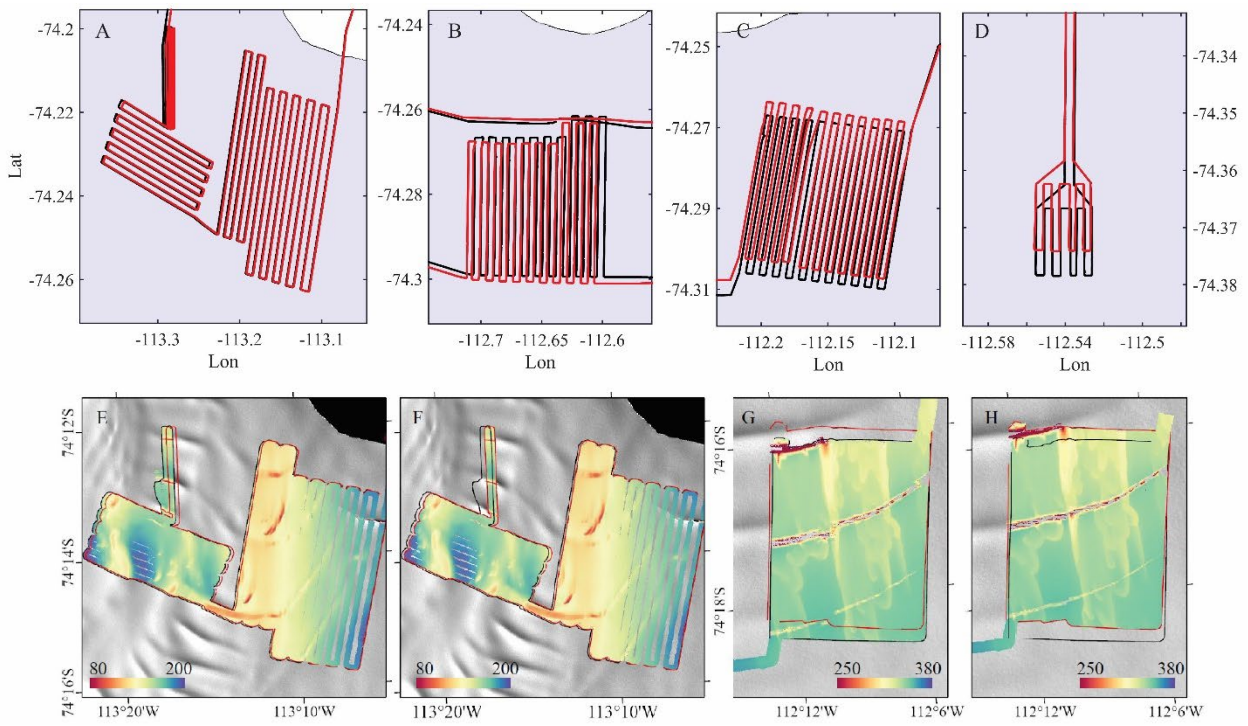

Figure S9: Post-processing of navigation. Top panels show the Autonomous Underwater Vehicle (AUV) path before (black) and after (red) post processing of navigation for (A) Maps W2 and W3 (B) Map C1 (C) Map E1 and (D) Map C2. Lower panels show the ice draft in maps W2, W3, and E1, overlaid on the Landsat-8 image on February 15, 2022 and gridded using the (E) raw navigation (F) post-processed navigation (G) raw navigation (H) post-processed navigation.

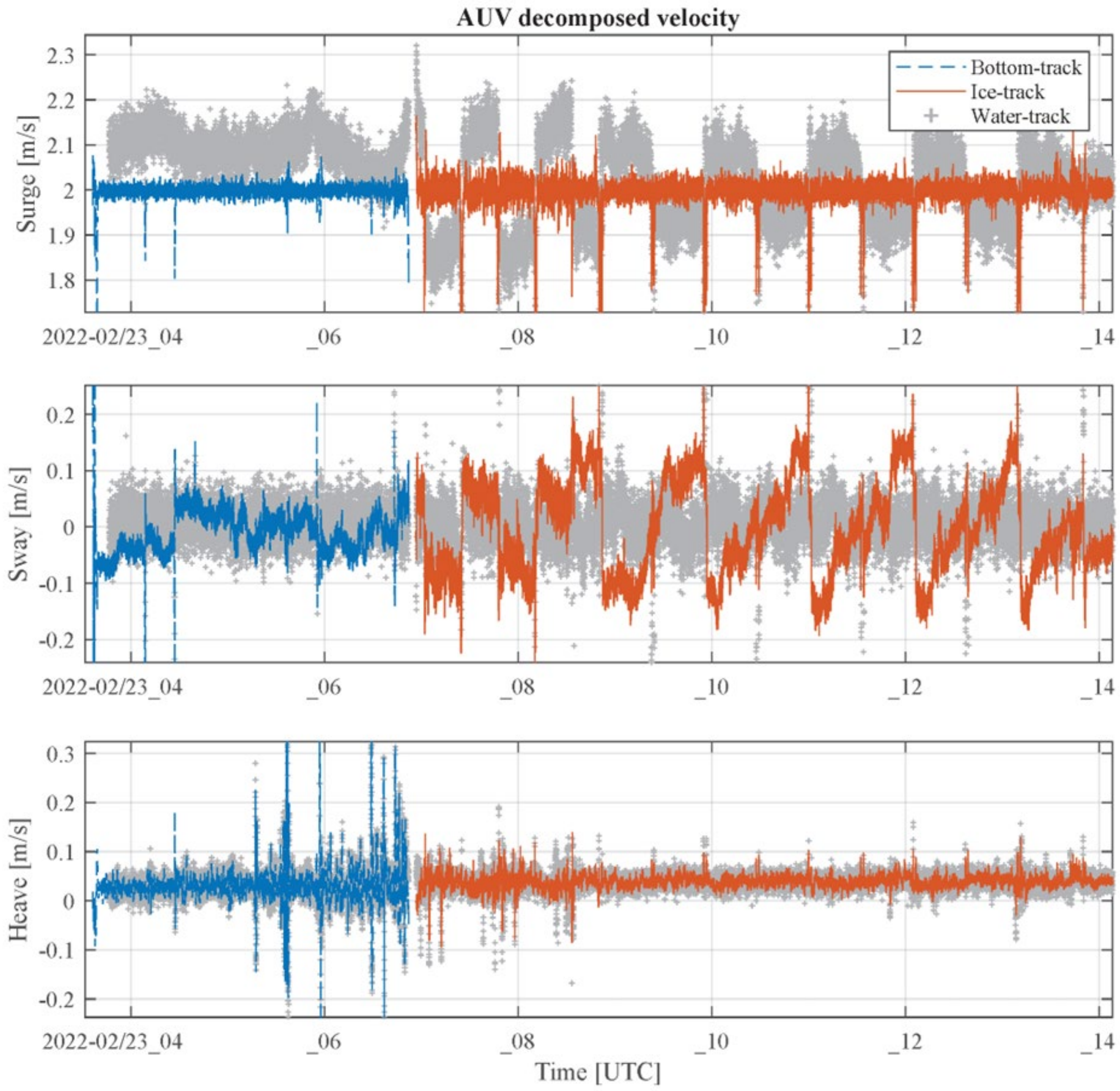

Figure S10: Transit to, and survey of, area W2. Autonomous Underwater Vehicle (AUV) velocities (body coordinate frame) relative to the bottom, ice, and water.

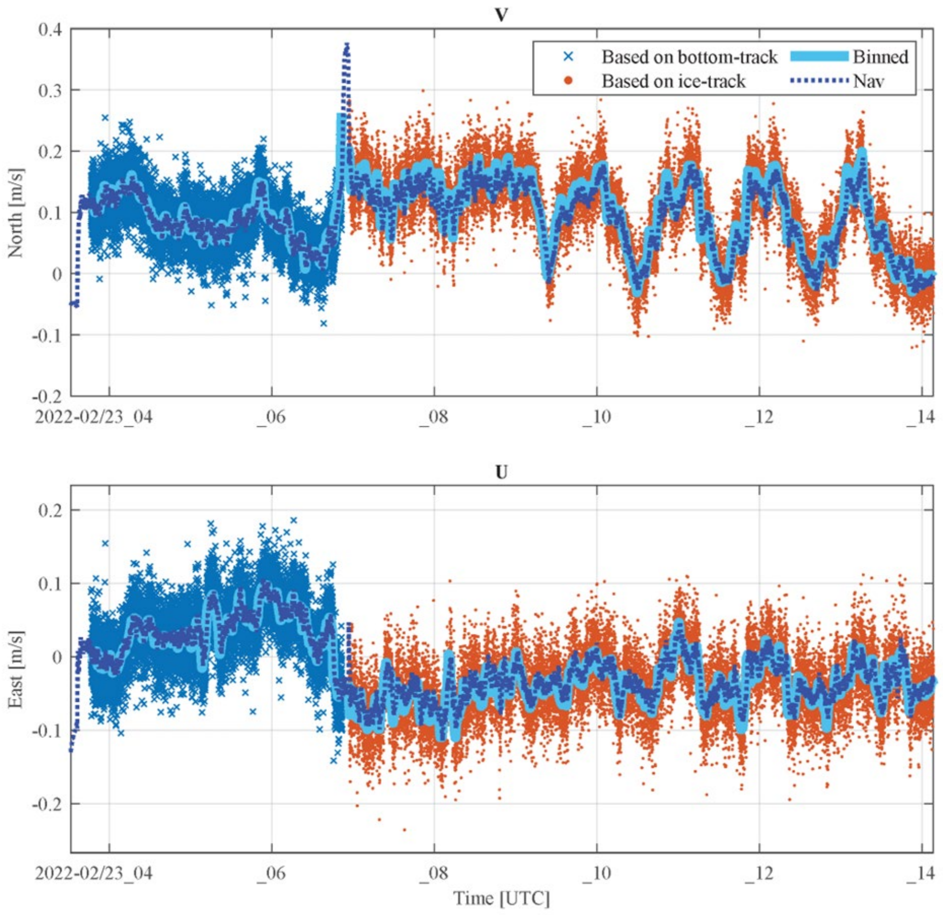

Figure S11. Estimated sea current from transit to, and survey of area W2. Data denoted "Binned" were used in the results section of this article. The sea current obtained and binned from the Acoustic Doppler Current Profiler (ADCP) data are consistent with the sea current estimated by the integrated inertial navigation system in the Autonomous Underwater Vehicle (AUV).

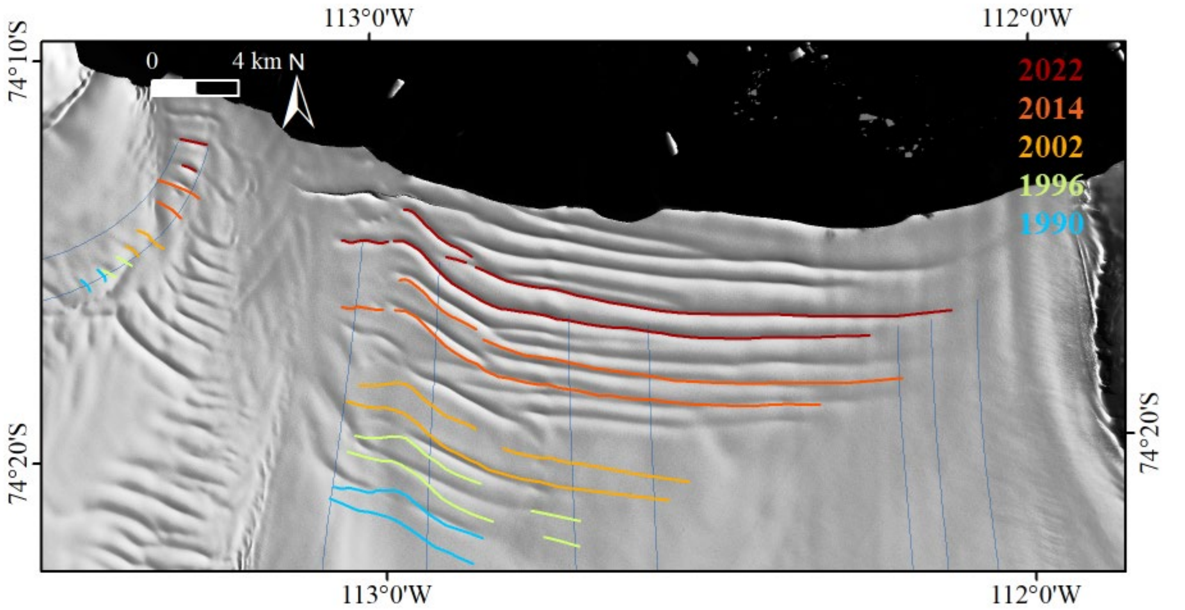

Figure S12: Fracture time series. Time series of typical fractures' locations on Dotson Ice Shelf, overlaid on the Sentinel-2 image on February 2, 2022. The colored lines indicate the shapes and locations of the fractures in 1990, 1996, 2002, 2014, and 2022, respectively. The thin blue lines show ice flowlines on the ice shelf, calculated based on the MEaSUREs InSAR-based ice velocity map(*73*).

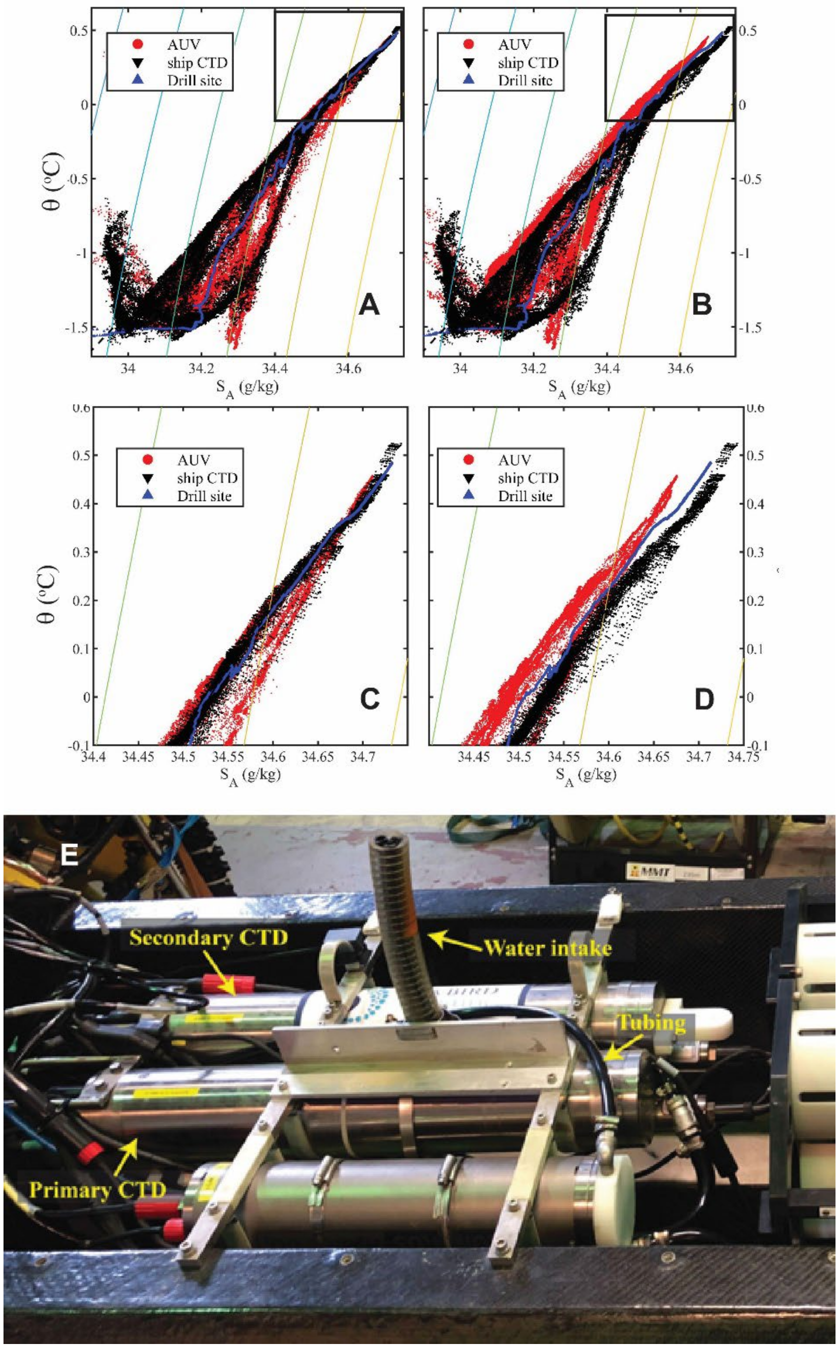

Figure S13: The Conductivity, Temperature, Depth (CTD) package in the Autonomous Underwater Vehicle (AUV). Plots of conservative temperature (Θ, ºC) versus absolute salinity (S_A, g kg^{-1}) for the AUV (red), ship (black) and borehole (blue) CTDs after (A,C) and before (B,D) offset correction. The black rectangles in (A) and (B) show the extent of the zoomed in area in (C) and (D). (e) Photograph of the CTD sensors mounted inside the hull

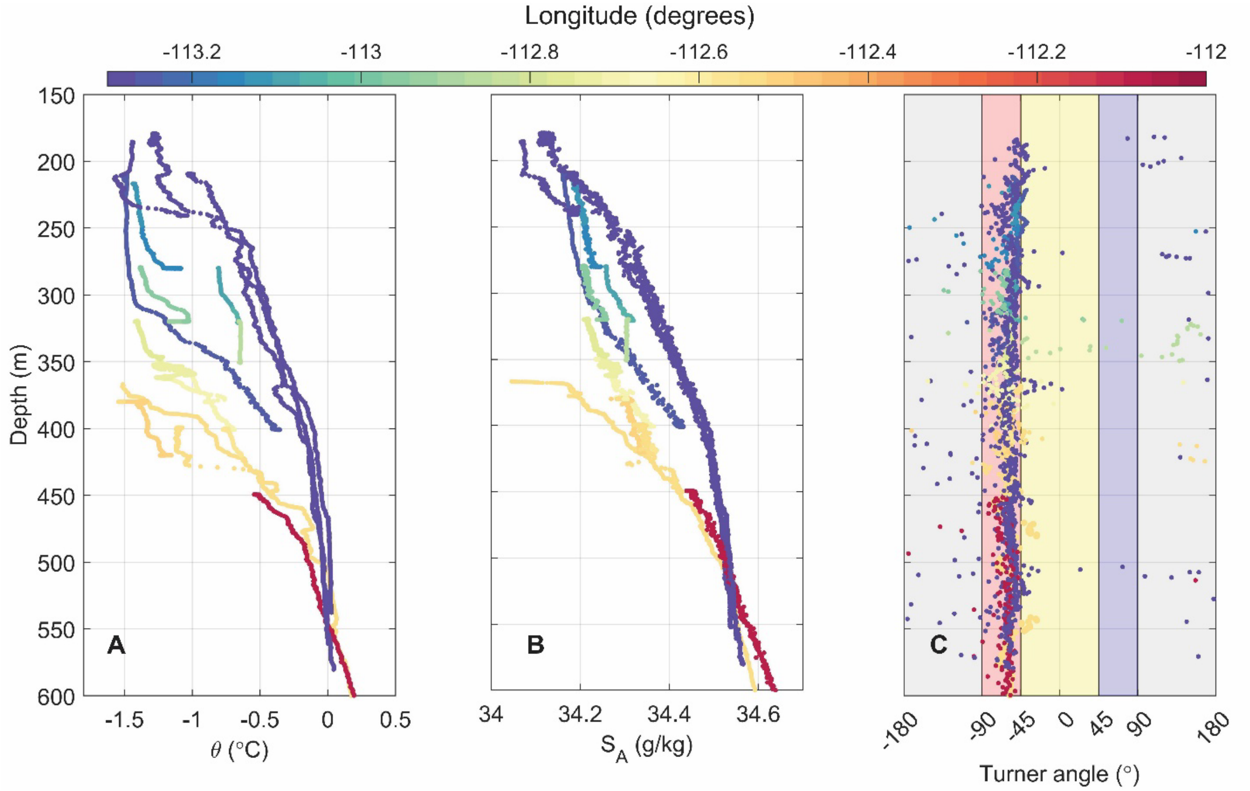

Figure S14. Turner angle and stratification for borehole data and Autonomous Underwater Vehicle (AUV) dives/ascents. Color corresponds to longitude (color bar). The temperature- and salinity profiles in (A) and (B) are not smoothed, and for (C) the data were smoothed using a 10 dbar running average before calculating the Turner angle.

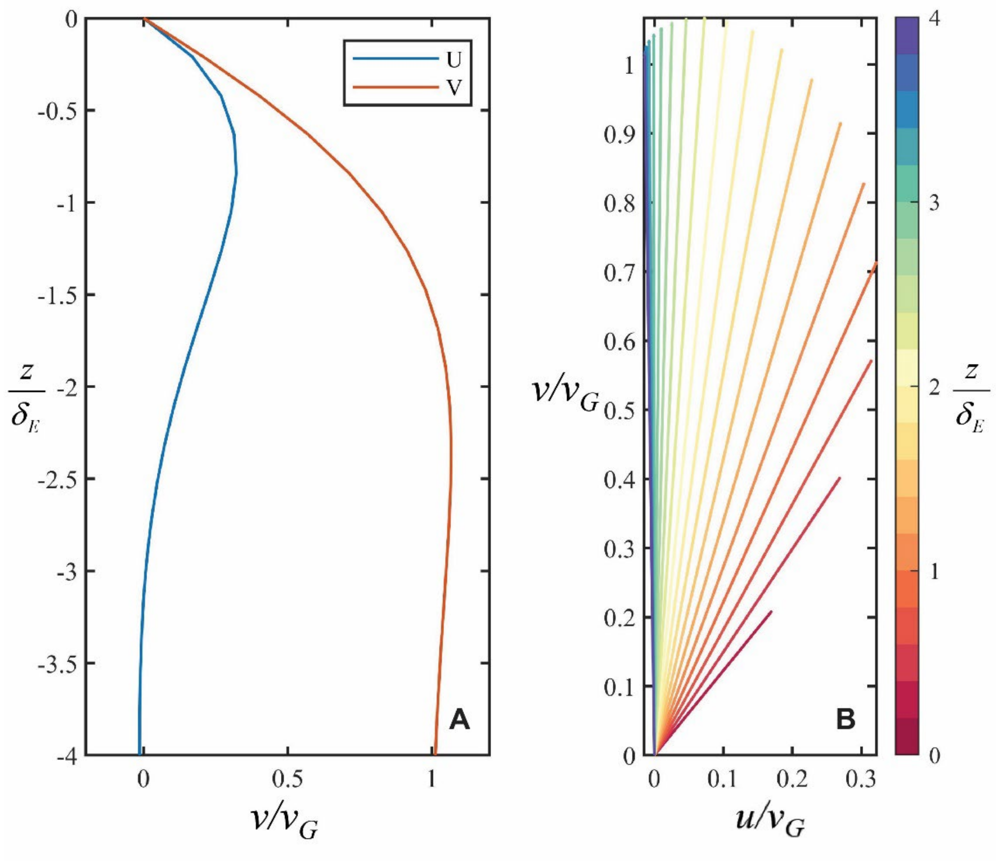

Figure S15: Ekman layer in the ice-ocean boundary layer. (A) Velocity components (normalized with v_G) as function of distance from the ice (normalized with the Ekman layer thickness δ_E). (B) Velocity vectors (normalized with v_G) for 20 different distances from the ice base, starting at z = 0 and ending at $4\delta_E$.

Table S1: List of Landsat images on which the fracture age estimates were based

Landsat mission	Path/row	Image acquisition date
8	009 113	February 15, 2022
8	008 113	November 20, 2021
8	008 113	January 20, 2021
8	010 113	December 1, 2020
8	007 113	February 28, 2020
8	008 113	November 15, 2019
8	009 113	January 22, 2019
8	008 113	November 12, 2018
8	008 113	February 13, 2018
8	010 113	November 7, 2017
8	008 113	January 25, 2017
8	010 113	November 4, 2016
8	009 113	January 30, 2016
8	009 113	October 26, 2015
8	010 113	February 3, 2015
8	010 113	October 14, 2014
8	009 113	February 9, 2014
8	009 113	November 5, 2013
7	009 113	February 14, 2013
7	009 113	November 10, 2012
7	009 113	January 24, 2011
7	008 113	December 16, 2010
7	007 113	February 8, 2010
7	009 113	December 20, 2009
7	009 113	February 19, 2009
7	007 113	December 19, 2008
7	010 113	January 23, 2008
7	008 113	November 6, 2007
7	009 113	December 12, 2006

7	009 113	February 24, 2005
7	007 113	February 8, 2004
7	008 113	November 24, 2002
7	009 113	November 28, 2001
7	164 131	December 9, 2000
7	010 113	January 1, 2000
5	010 113	February 1, 1997
4	008 113	January 23, 1990

Table S2. Source water conservative temperature (ϴ, ºC) and absolute salinity (SA, g kg^{-1}) for the water masses used to calculate the meltwater fraction (1)

Name	Suffix	ϴ (ºC)	SA (g kg^{-1})
Modified circumpolar deep water	MCDW	0.5	34.73
Winter Water	WW	-1.86	34.25
Melt water	MW	-90.8	0

Table S3: Melt rates based on shear-driven parameterization. Melt rates based on the 3-equation parameterization based on the observed conservative temperature Θ, absolute salinity S_A and velocity u below the ice. The table shows the range of values using the 3-equation parameterization with the standard heat and salt transfer coefficients (*14*).

**Θ (°C)**	**S_A (g kg^{-1})**	**u (m s^{-1})**	**m (m yr^{-1})**
-1.7	34.05	0.15	3.2
-1.7	34.15	0.15	3.2
-1.7	34.05	0.25	5.3
-1.7	34.15	0.25	5.4
-1.2	34.05	0.15	9.2
-1.2	34.15	0.15	9.3
-1.2	34.05	0.25	15.4
-1.2	34.15	0.25	15.5

REFERENCES AND NOTES

1. R. D. Larter, Basal melting, roughness and structural integrity of ice shelves. *Geophys. Res. Lett.* **49**, e2021GL097421 (2022).

2. H. D. Pritchard, S. R. M. Ligtenberg, H. A. Fricker, D. G. Vaughan, M. R. Van Den Broeke, L. Padman, Antarctic ice-sheet loss driven by basal melting of ice shelves. *Nature* **484**, 502–505 (2012).

3. T. K. Dupont, R. B. Alley, Assessment of the importance of ice-shelf buttressing to ice-sheet flow. *Geophys. Res. Lett.* **32**, 1–4 (2005).

4. C. Schoof, Ice sheet grounding line dynamics: Steady states, stability, and hysteresis. *Case Rep. Med.* **112** (2007).

5. D. T. Bett, A. T. Bradley, C. R. Williams, P. R. Holland, R. J. Arthern, D. N. Goldberg, Coupled ice-ocean interactions during the future retreat of West Antarctic ice streams. *The Cryosphere*, **18**, 2653-2675 (2024).

6. P. E. D. Davis, A. Jenkins, K. W. Nicholls, P. V. Brennan, E. P. Abrahamsen, K. J. Heywood, P. Dutrieux, K. H. Cho, T. W. Kim, Variability in basal melting beneath Pine Island Ice Shelf on weekly to monthly timescales. *J. Geophys. Res. Oceans* **123**, 8655–8669 (2018).

7. C. L. Stewart, P. Christoffersen, K. W. Nicholls, M. J. M. Williams, J. A. Dowdeswell, Basal melting of Ross Ice Shelf from solar heat absorption in an ice-front polynya. *Nat. Geosci.* **12**, 435–440 (2019).

8. L. Herraiz-Borreguero, R. Coleman, I. Allison, S. R. Rintoul, M. Craven, G. D. Williams, Circulation of modified Circumpolar Deep Water and basal melt beneath the Amery Ice Shelf, East Antarctica, *J. Geophys. Res. Oceans* **120**, 3098–3112 (2015).

9. P. E. D. Davis, K. W. Nicholls, D. M. Holland, B. E. Schmidt, P. Washam, K. L. Riverman, R. J. Arthern, I. Vaňková, C. Eayrs, J. A. Smith, P. G. D. Anker, A. D. Mullen, D. Dichek, J. D. Lawrence, M. M. Meister, E. Clyne, A. Basinski-Ferris, E. Rignot, B. Y. Queste, L. Boehme, K.

J. Heywood, S. Anandakrishnan, K. Makinson, Suppressed basal melting in the eastern Thwaites Glacier grounding zone. *Nature* **614**, 479–485 (2023).

10. E. Lambert, A. Jüling, R. S. W. Van De Wal, P. R. Holland, Modelling Antarctic ice shelf basal melt patterns using the one-layer Antarctic model for dynamical downscaling of ice-ocean exchanges (LADDIE v1.0). *Cryosphere* **17**, 3203–3228 (2023).

11. A. Malyarenko, A. J. Wells, P. J. Langhorne, N. J. Robinson, M. J. M. Williams, K. W. Nicholls, A synthesis of thermodynamic ablation at ice–ocean interfaces from theory, observations and models. *Ocean Model (Oxf)* **154**, 101692 (2020).

12. M. Rosevear, B. Galton-Fenzi, C. Stevens, Evaluation of basal melting parameterisations using in situ ocean and melting observations from the Amery Ice Shelf, East Antarctica, *Ocean Sci.* **18**, 1109–1130 (2022).

13. C. B. Begeman, S. M. Tulaczyk, O. J. Marsh, J. A. Mikucki, T. P. Stanton, T. O. Hodson, M. R. Siegfried, R. D. Powell, K. Christianson, M. A. King, Ocean stratification and low melt rates at the Ross Ice Shelf Grounding Zone. *J. Geophys. Res. Oceans* **123**, 7438–7452 (2018).

14. A. Jenkins, Convection-driven melting near the grounding lines of ice shelves and tidewater glaciers. *J. Phys. Oceanogr.* **41**, 2279–2294 (2011).

15. M. Dinniman, X. Asay-Davis, B. Galton-Fenzi, P. Holland, A. Jenkins, R. Timmermann, Modeling ice shelf/ocean interaction in Antarctica: A review. *Oceanography* **29**, 144–153 (2016).

16. S. Kimura, K. W. Nicholls, E. Venables, Estimation of ice shelf melt rate in the presence of a thermohaline staircase. *J. Phys. Oceanogr.* **45**, 133–148 (2015).

17. L. Middleton, C. A. Vreugdenhil, P. R. Holland, J. R. Taylor, Numerical simulations of melt-driven double-diffusive fluxes in a turbulent boundary layer beneath an ice shelf. *J. Phys. Oceanogr.* **51**, 403–418 (2021).

18. S. S. Pegler, M. S. Davies Wykes, Shaping of melting and dissolving solids under natural convection. *J. Fluid Mech.* **900**, 35–36 (2020).

19. I. Vaňková, K. W. Nicholls, Ocean variability beneath the Filchner-Ronne Ice Shelf inferred from basal melt rate time series. *J. Geophys. Res. Oceans* **127**, JC018879 (2022).

20. M. G. Rosevear, B. Gayen, B. K. Galton-Fenzi, The role of double-diffusive convection in basal melting of Antarctic ice shelves. *Proc. Natl. Acad. Sci. U.S.A.* **118**, e2007541118 (2021).

21. J. S. Na, P. E. D. Davis, B. H. Kim, E. K. Jin, W. S. Lee, Ice shelf water structure beneath the Larsen C Ice Shelf in Antarctica. *Geophys. Res. Lett.* **50**, GL104088 (2023).

22. R. H. Watkins, J. N. Bassis, M. D. Thouless, Roughness of ice shelves is correlated with basal melt rates. *Geophys. Res. Lett.* **48**, e2021GL094743 (2021).

23. P. Dutrieux, C. Stewart, A. Jenkins, K. W. Nicholls, H. F. J. Corr, E. Rignot, K. Steffen, Basal terraces on melting ice shelves. *Geophys. Res. Lett.* **41**, 5506–5513 (2014).

24. K. W. Nicholls, E. P. Abrahamsen, J. J. H. Buck, P. A. Dodd, C. Goldblatt, G. Griffiths, K. J. Heywood, N. E. Hughes, A. Kaletzky, S. D. Mcphail, N. W. Millard, K. I. C. Oliver, J. Perrett, M. R. Price, C. J. Pudsey, K. Saw, K. Stansfield, M. J. Stott, P. Wadhams, A. T. Webb, J. P. Wilkinson, Measurements beneath an Antarctic ice shelf using an autonomous underwater vehicle. *Geophys. Res. Lett.* **33**, 2–5 (2006).

25. B. E. Schmidt, P. Washam, P. E. D. Davis, K. W. Nicholls, D. M. Holland, J. D. Lawrence, K. L. Riverman, J. A. Smith, A. Spears, D. J. G. Dichek, A. D. Mullen, E. Clyne, B. Yeager, P. Anker, M. R. Meister, B. C. Hurwitz, E. S. Quartini, F. E. Bryson, A. Basinski-Ferris, C. Thomas, J. Wake, D. G. Vaughan, S. Anandakrishnan, E. Rignot, J. Paden, K. Makinson, Heterogeneous melting near the Thwaites Glacier grounding line. *Nature* **614**, 471–478 (2023).

26. K. E. Alley, T. A. Scambos, R. B. Alley, N. Holschuh, Troughs developed in ice-stream shear margins precondition ice shelves for ocean-driven breakup. *Sci. Adv.* **5**, eaax2215 (2019).

27. K. E. Alley, T. A. Scambos, M. R. Siegfried, H. A. Fricker, Impacts of warm water on Antarctic ice shelf stability through basal channel formation. *Nat. Geosci.* **9**, 290–293 (2016).

28. N. Gourmelen, D. N. Goldberg, K. Snow, S. F. Henley, R. G. Bingham, S. Kimura, A. E. Hogg, A. Shepherd, J. Mouginot, J. T. M. Lenaerts, S. R. M. Ligtenberg, W. J. van de Berg, Channelized melting drives thinning under a rapidly melting Antarctic ice shelf. *Geophys. Res. Lett.* **44**, 9796–9804 (2017).

29. S. Weady, J. Tong, A. Zidovska, L. Ristroph, Anomalous convective flows carve pinnacles and scallops in melting ice. *Phys. Rev. Lett.* **128**, 044502 (2022).

30. J. D. Lawrence, P. M. Washam, C. Stevens, C. Hulbe, H. J. Horgan, G. Dunbar, T. Calkin, C. Stewart, N. Robinson, A. D. Mullen, M. R. Meister, B. C. Hurwitz, E. Quartini, D. J. G. Dichek, A. Spears, B. E. Schmidt, Crevasse refreezing and signatures of retreat observed at Kamb Ice Stream grounding zone. *Nat. Geosci.* **16**, 238–243 (2023).

31. P. Washam, J. D. Lawrence, C. L. Stevens, C. L. Hulbe, H. J. Horgan, N. J. Robinson, C. L. Stewart, A. Spears, E. Quartini, B. Hurwitz, M. R. Meister, A. D. Mullen, D. J. Dichek, F. Bryson, B. E. Schmidt, Direct observations of melting, freezing, and ocean circulation in an ice shelf basal crevasse. *Sci. Adv.* **9**, eadi7638 (2023).

32. S. Adusumilli, H. A. Fricker, B. Medley, L. Padman, M. R. Siegfried, Interannual variations in meltwater input to the Southern Ocean from Antarctic ice shelves. *Nat. Geosci.* **13**, 616–620 (2020).

33. D. A. Lilien, I. Joughin, B. Smith, D. E. Shean, Changes in flow of Crosson and Dotson ice shelves, West Antarctica, in response to elevated melt. *Cryosphere* **12**, 1415–1431 (2018).

34. A. S. P. Zinck, B. Wouters, E. Lambert, S. Lhermitte, Unveiling spatial variability within the Dotson Melt Channel through high-resolution basal melt rates from the Reference Elevation Model of Antarctica. *Cryosphere* **17**, 3785–3801 (2023).

35. A. K. Wåhlin, X. Yuan, G. Björk, C. Nohr, Inflow of warm circumpolar deep water in the Central Amundsen Shelf*. *J. Phys. Oceanogr.* **40**, 1427–1434 (2010).

36. H. W. Yang, T. W. Kim, P. Dutrieux, A. K. Wåhlin, A. Jenkins, H. K. Ha, C. S. Kim, K. H. Cho, T. Park, S. H. Lee, Y. K. Cho, Seasonal variability of ocean circulation near the Dotson Ice Shelf, Antarctica, *Nat. Commun.* **13**, 1138 (2022).

37. T. Miles, S. H. Lee, A. Wåhlin, H. K. Ha, T.-W. W. Kim, K. M. Assmann, O. Schofield, Glider observations of the Dotson Ice Shelf outflow. *Deep-Sea Res. II Top. Stud. Oceanogr.* **123**, 16–29 (2015).

38. A. K. Wåhlin, N. Steiger, E. Darelius, K. M. Assmann, M. S. Glessmer, H. K. Ha, L. Herraiz-Borreguero, C. Heuzé, A. Jenkins, T. W. Kim, A. K. Mazur, J. Sommeria, S. Viboud, Ice front blocking of ocean heat transport to an Antarctic ice shelf. *Nature* **578**, 568–571 (2020).

39. A. G. Stubblefield, M. G. Wearing, C. R. Meyer, Linear analysis of ice-shelf topography response to basal melting and freezing. *Royal Soc.* **479** (2023). 10.1098/rspa.2023.0290

40. A. Jenkins, D. Shoosmith, P. Dutrieux, S. Jacobs, T. W. Kim, S. H. Lee, H. K. Ha, S. Stammerjohn, West Antarctic Ice Sheet retreat in the Amundsen Sea driven by decadal oceanic variability. *Nat. Geosci.* **11**, 733–738 (2018).

41. C. S. Kim, T. W. Kim, K. H. Cho, H. K. Ha, S. H. Lee, H. C. Kim, J. H. Lee, Variability of the Antarctic Coastal Current in the Amundsen Sea. *Estuar. Coast. Shelf Sci.* **181**, 123–133 (2016).

42. E. Randall-Goodwin, M. P. Meredith, A. Jenkins, P. L. Yager, R. M. Sherrell, E. P. Abrahamsen, R. Guerrero, X. Yuan, R. A. Mortlock, K. Gavahan, A. C. Alderkamp, H. Ducklow, R. Robertson, S. E. Stammerjohn, Freshwater distributions and water mass structure in the Amundsen Sea Polynya region, Antarctica, *Elementa* **3**, 65 (2015).

43. A. Jenkins, A simple model of the ice shelf–ocean boundary layer and current. *J. Phys. Oceanogr.* **46**, 1785–1803 (2016).

44. B. Ruddick, A practical indicator of the stability of the water column to double-diffusive activity. *Deep Sea Res. Part A. Oceanogr. Res. Papers* **30**, 1105–1107 (1983).

45. S. S. Jacobs, H. E. Huppert, G. Holdsworth, D. J. Drewry, Thermohaline steps induced by melting of the Erebus Glacier Tongue. *J. Geophys. Res.* **86**, 6547–6555 (1981).

46. M. G. Rosevear, B. Gayen, B. K. Galton-Fenzi, Regimes and transitions in the basal melting of Antarctic ice shelves. *J. Phys. Oceanogr.* **52**, 2589–2608 (2022).

47. L. Middleton, P. E. D. Davis, J. R. Taylor, K. W. Nicholls, Double diffusion as a driver of turbulence in the stratified boundary layer beneath George VI Ice Shelf. *Geophys. Res. Lett.* **49**, e2021GL096119 (2022).

48. T. Radko, Thermohaline-shear instability. *Geophys. Res. Lett.* **46**, 822–832 (2019).

49. A. Fildani, Submarine canyons: A brief review looking forward. *Geology* **45**, 383–384 (2017).

50. M. Poinelli, M. Schodlok, E. Larour, M. Vizcaino, R. Riva, Can rifts alter ocean dynamics beneath ice shelves? *Cryosphere* **17**, 2261–2283 (2023).

51. J. N. Bassis, Y. Ma, Evolution of basal crevasses links ice shelf stability to ocean forcing. *Earth Planet. Sci. Lett.* **409**, 203–211 (2015).

52. G. D. Raithby, K. G. T. Hollands, T. E. Unny, Analysis of heat transfer by natural convection across vertical fluid layers. *J. Heat Transfer* **99**, 287–293 (1977).

53. S. Van Der Walt, J. L. Schönberger, J. Nunez-Iglesias, F. Boulogne, J. D. Warner, N. Yager, E. Gouillart, T. Yu, scikit-image: Image processing in Python. *PeerJ* **2**, e453 (2014).

54. W. E. Lorensen, H. E. Cline, Marching cubes: A high resolution 3D surface construction algorithm. *Comput. Graphics (ACM)* **21**, 163–169 (1987).

55. Ø. Hegrenæs, E. Berglund, “Doppler water-track aided inertial navigation for autonomous underwater vehicle” in *OCEANS ‘09 IEEE Bremen: Balancing Technology with Future Needs* (IEEE, 2009), doi: 10.1109/OCEANSE.2009.5278307.

56. Ø. Hegrenaes, O. Hallingstad, Model-aided INS with sea current estimation for robust underwater navigation. *IEEE J. Oceanic Eng.* **36**, 316–337 (2011).

57. Ø. Hegrenæs, K. Gade, O. K. Hagen, P. E. Hagen, “Underwater transponder positioning and navigation of autonomous underwater vehicles” in *Proceedings of the MTS/IEEE Oceans Conference and Exhibition* (IEEE, 2009), https://ieeexplore.ieee.org/document/5422358).

58. K. Gade, NAVLAB, a generic simulation and post-processing tool for navigation. *Eur. J. Navig. Sci.* **2,** 1-9 (2004).

59. S. Erofeeva, L. Padman, S. Howard, “Tide Model Driver (TMD) version 2.5, Toolbox for Matlab” (2020); https://github.com/EarthAndSpaceResearch/TMD_Matlab_Toolbox_v2.5.

60. T. J. McDougall, P. M. Barker, *Getting Started with TEOS-10 and the Gibbs Seawater (GSW) Oceanographic Toolbox, 28pp, SCOR/IAPSO WG127,* (2011).

61. E. Firing, J. M. Hummon, T. K. Chereskin, Improving the quality and accessibility of current profile measurements in the southern ocean. *Oceanography* **25**, 164–165 (2012).

62. T. A. Jordan, D. Porter, K. Tinto, R. Millan, A. Muto, K. Hogan, R. D. Larter, A. G. C. Graham, J. D. Paden, New gravity-derived bathymetry for the Thwaites, Crosson, and Dotson ice shelves revealing two ice shelf populations. *Cryosphere* **14**, 2869–2882 (2020).

63. H. Gade, Melting of ice in sea water: A primitive model with application to the Antarctic ice shelf and icebergs. *J. Phys. Oceanogr.* **9**, 189–198 (1979).

64. A. Jenkins, The impact of melting ice on ocean waters. *J. Phys. Oceanogr.* **29**, 2370–2381 (1999).

65. A. K. Wåhlin, G. Walin, Downward migration of dense bottom currents. *Environ. Fluid Mech.* **1**, 257–279 (2001).

66. I. Howat, C. Porter, M.-J. Noh, E. Husby, S. Khuvis, E. Danish, K. Tomko, J. Gardiner, A. Negrete, B. Yadav, J. Klassen, C. Kelleher, M. Cloutier, J. Bakker, J. Enos, G. Arnold, G. Bauer, P. Morin, The Reference Elevation Model of Antarctica - Mosaics, Version 2. doi: 10.7910/DVN/EBW8UC (2022).

67. R. Bindschadler, H. Choi, High-resolution Image-derived Grounding and Hydrostatic Lines for the Antarctic Ice Sheet, Version 1 | National Snow and Ice Data Center. https://doi.org/10.7265/N56T0JK2 (2011).

68. E. Rignot, J. Mouginot, B. Scheuchl, *MEaSUREs Antarctic Grounding Line from Differential Satellite Radar Interferometry, Version 2 [Data Set]*, NASA National Snow and Ice Data Center Distributed Active Archive Center (2016).

69. P. Milillo, E. Rignot, P. Rizzoli, B. Scheuchl, J. Mouginot, J. L. Bueso-Bello, P. Prats-Iraola, L. Dini, Rapid glacier retreat rates observed in West Antarctica. *Nature* **15**, 48–53 (2022).

70. MEaSUREs BedMachine Antarctica, Version 3 | National Snow and Ice Data Center. https://nsidc.org/data/nsidc-0756/versions/3.

71. J. Mouginot, E. Rignot, B. Scheuchl, Continent-wide, interferometric SAR phase, mapping of Antarctic ice velocity. *Geophys. Res. Lett.* **46**, 9710–9718 (2019).
